# Cutaneous Lyme borreliosis: Guideline of the German Dermatology Society

**DOI:** 10.3205/000348

**Published:** 2025-10-09

**Authors:** Heidelore Hofmann, Volker Fingerle, Sebastian Rauer, Klaus-Peter Hunfeld, Hans-Iko Huppertz, Andreas Krause, Consensus group

**Affiliations:** 1 Klinik für Dermatologie und Allergologie der TU München, Munich, Germany; 2 Nationales Referenzzentrum für Borrelien, Bayerisches Landesamt für Gesundheit und Lebensmittelsicherheit (LGL), Oberschleißheim, Germany; 3 Neurologische Universitätsklinik, Freiburg, Germany; 4 Zentralinstitut für Labormedizin, Mikrobiologie & Krankenhaushygiene, Krankenhaus Nordwest, Frankfurt, Germany; 5 Professor-Hess-Kinderklinik Klinikum Bremen-Mitte, Bremen, Germany; 6 Immanuel Krankenhaus Berlin, Germany

## Abstract

The current S2k guideline “kutane Borreliose” has been updated in accordance with the methodological standards of the “Arbeitsgemeinschaft der Wissenschaftlichen Medizinischen Fachgesellschaften e.V.”. It has received consensus from 18 AWMF member societies, the Robert Koch Institute, the “Paul-Ehrlich-Gesellschaft für Infektionstherapie e.V.”, “Instand e.V. – Gesellschaft zur Förderung der Qualitätssicherung in medizinischen Laboratorien”, the “Deutsche Borreliose-Gesellschaft” and the two German patient organisations “Borreliose und FSME Bund Deutschland” and “Aktionsbündnis gegen zeckenübertragene Infektionen Deutschland e.V.”.

This guideline of the German Dermatology Society primarily focuses on the diagnosis and treatment of cutaneous manifestations of Lyme borreliosis and is directed at physicians in private practices and clinics who treat Lyme borreliosis. Objectives of this guideline are recommendations for confirming a clinical diagnosis, recommendations for a stage-related laboratory diagnosis and recommendations for the treatment of the different manifestations erythema migrans, multiple erythemata migrantia, borrelial lymphocytoma, and acrodermatitis chronica atrophicans.

The current update of the guideline incorporates the international literature on cutaneous manifestations of Lyme borreliosis up to 2022. There have been no significant changes in diagnosis and treatment. The Cochrane network analysis on the treatment of erythema migrans has only shown that, in addition to doxycycline and amoxicillin, oral penicillin V is equally effective. A Slovenian analysis of patients with acrodermatitis chronica atrophicans over the past 30 years has shown a decrease in the incidence of constitutional symptoms and atrophy, likely due to improved early detection. In addition, an information sheet for patients containing recommendations for the prevention of Lyme borreliosis is attached to the guideline.

## Abstract (de)

Die aktuelle S2k-Leitlinie „kutane Borreliose“ wurde entsprechend den methodischen Standards der „Arbeitsgemeinschaft der Wissenschaftlichen Medizinischen Fachgesellschaften e.V.“ aktualisiert. Sie wurde interdisziplinär von 18 AWMF-Mitgliedsgesellschaften, dem Robert Koch-Institut, der „Paul-Ehrlich-Gesellschaft für Infektionstherapie“, der „Instand e.V. – Gesellschaft zur Förderung der Qualitätssicherung in medizinischen Laboratorien“, der „Deutschen Borreliose-Gesellschaft“ und den beiden deutschen Patientenorganisationen „Borreliose und FSME Bund Deutschland“ und „Aktionsbündnis gegen zeckenübertragene Infektionen Deutschland e.V.“ konsentiert.

Diese Leitlinie der Deutschen Dermatologischen Gesellschaft befasst sich primär mit der Diagnostik und Therapie kutaner Manifestationen der Lyme-Borreliose und richtet sich an niedergelassene Ärzte und Kliniker, die Lyme-Borreliose behandeln. Ziele dieser Leitlinie sind Empfehlungen zur Sicherung der klinischen Diagnose, Empfehlungen zur stadienbezogenen Labordiagnostik sowie Empfehlungen zur Therapie der verschiedenen Manifestationen Erythema migrans, Multiple Erythemata migrantia, Borrelien-Lymphozytom und Acrodermatitis chronica atrophicans.

Die vorliegende Aktualisierung der Leitlinie berücksichtigt die internationale Literatur zu kutanen Manifestationen der Lyme-Borreliose bis 2022. Es gab keine wesentlichen Änderungen in Diagnostik und Therapie. Die Cochrane-Netzwerkanalyse zur Behandlung des Erythema migrans hat lediglich gezeigt, dass orales Penicillin V neben Doxycyclin und Amoxicillin ebenso wirksam ist. Eine slowenische Analyse von Patienten mit Acrodermatitis chronica atrophicans über die letzten 30 Jahre zeigte einen Rückgang der Inzidenz von Allgemeinsymptomen und Atrophie, vermutlich aufgrund einer verbesserten Früherkennung. Darüber hinaus ist der Leitlinie ein Informationsblatt für Patienten mit Empfehlungen zur Prävention der Lyme-Borreliose beigefügt.

## Preamble

This guideline primarily focuses on the diagnosis and treatment of cutaneous manifestations of Lyme borreliosis. 

It was approved in 2016 by 22 interdisciplinary medical societies and 2 patient organisations. Since then, a systematic literature review and evaluation has been conducted and published by the German Cochrane Centre, Freiburg (Cochrane Germany) for the further development to stage S3 for the treatment of erythema migrans [1].

In 2018, the same interdisciplinary guideline group published the guideline ‘Neuroborreliosis’, AWMF register number 030-071, development stage S3 [2].

### What’s new?

The updated version of this guideline incorporates international publications on cutaneous manifestations of Lyme borreliosis up to 2022.

There have been no significant changes regarding diagnosis and treatment. Cochrane’s network meta-analysis on the treatment of erythema migrans has shown that oral penicillin V is just as effective as doxycycline and amoxicillin. A Slovenian study of patients with acrodermatitis chronica atrophicans, carried out over the last 30 years, has found that constitutional symptoms and atrophy are becoming less frequent [3], most likely due to improvements in early detection. 

### Synonyms

Cutaneous borreliosis, cutaneous manifestations of Lyme borreliosis, skin borreliosis, cutaneous Lyme borreliosis, cutaneous Lyme disease

### Search terms

*Borrelia burgdorferi* infection, hard-bodied tick borreliosis, Lyme disease, cutaneous Lyme borreliosis, erythema migrans disease, erythema migrans, erythema chronicum migrans, lymphadenosis cutis benigna, borrelial lymphocytoma, multiple erythemata migrantia, multiple erythema migrans, acrodermatitis chronica atrophicans

ICD-10-No.: A69.2, L90.4 

AWMF Guideline Register No. 013/044

Development stage: S2k

## List of abbreviations

- ACA: Acrodermatitis chronica atrophicans
- BL: Borrelial lymphocytoma
- DD: Differential diagnosis
- d: Day
- EM: Erythema migrans
- ECM: Erythema chronicum migrans
- i.v.: Intravenous
- BW: Body weight
- LB: Lyme borreliosis
- LTT: Lymphozyte transformation test
- MEM: Multiple erythemata migrantia
- MiQ: Quality standards in microbiological diagnosis of infections
- NAT: Nucleic acid amplification techniques
- NSAR: Nonsteroidal anti-inflammatory drugs
- PCR: Polymerase chain reaction
- p.o. : Per os
- PPI: Proton pump inhibitor
- RCT: Randomised controlled trial
- SNRI: Serotonin norepinephrine reuptake inhibitor

## 1 Introduction

Lyme borreliosis is the infectious disease most frequently transmitted by ticks in Europe. The Borrelia migrate from the hard-bodied tick *Ixodes ricinus* into the bite wound during the act of blood feeding. There the Borrelia are either killed off by the (non-specific, innate) immune system, or a localised infection occurs which leads to illness in a small percentage of those infected. Most often the skin becomes inflamed, typically in the form of erythema migrans or, seldom, as borrelial lymphocytoma. In the course of the infection, the Borrelia can disseminate and attack various organs. They primarily affect the skin, joints and nervous system. Acrodermatitis chronica atrophicans can develop as a chronic or late-stage skin infection. 

### 1.1 Target group

This guideline is directed at physicians in private practices and clinics that treat Lyme borreliosis. 

### 1.2 Objectives of this guideline

- Recommendations for confirming a clinical diagnosis
- Recommendations for stage-appropriate laboratory testing: serological detection of IgM and IgG Borrelia antibodies using the 2-step ELISA/immunoblot process; prudent use of procedures for molecular and culture-based diagnostic testing
- Treatment of localised, early-stage infections (erythema migrans, erythema chronicum migrans and Borrelia lymphocytoma)
- Treatment of disseminated early infections (multiple erythemata migrantia, flu-like symptoms)
- Treatment of late-stage infections (acrodermatitis chronica without neurological manifestations)
- Treatment of late-stage infections (acrodermatitis chronica with neurological manifestations)
- Prevention of Lyme borreliosis
- Recommendations for observing tick bites
- Information sheet for patients (Annex 1 in Attachment 1)

### 1.3 Participating medical societies and patient organisations

#### Steering group

**Led by:**

Prof Dr med. Heidelore Hofmann – coordinator 

German Dermatology Society (DDG)

Dr med. Volker Fingerle – coordinator

German Society for Hygiene and Microbiology (DGHM) 

Prof Dr med. Sebastian Rauer

German Society of Neurology (DGN) – coordinator 

Deputy Dr Stefan Kastenbauer

German Society of Neurology (DGN)

Prof Dr med. Hans-Iko Huppertz

German Society of Paediatrics and Adolescent Medicine (DGKJ) and

German Society of Paediatric Infectiology (DGPI) 

Prof Dr med. Klaus-Peter Hunfeld

German United Society of Clinical Chemistry and Laboratory Medicine and 

INSTAND e.V. 

Prof Dr med. Andreas Krause

German Society for Rheumatology and Clinical Immunology (DGRh)

Prof Dr med. Bernd Salzberger

German Society of Infectious Diseases (DGI) 

#### Consensus group

Prof Dr med. Karl Bechter

The German Association of Psychiatry, Psychotherapy and Psychosomatics (DGPPN) 

Dr jur. Astrid Breinlinger

Borreliosis and FSME Association Germany (BFBD)

Ursula Dahlem 

Action Alliance Against Tick-Borne Infections Germany (OnLyme-Aktion)

Prof Dr med. Michael H. Freitag 

German Society for Occupational and Environmental Medicine (DEGAM) 

PD Dr med. Gudrun Gossrau 

German Pain Society 

Prof Dr med. Gerd Gross 

Paul Ehrlich Society for Chemotherapy (PEG) 

Prof Dr med. Constanze Hausteiner-Wiehle

German Society of Psychosomatic Medicine and Medical Psychotherapy (DGPM) and the 

German College of Psychosomatic Medicine (DKPM) 

Prof Dr med. Rainer Müller 

German Society of Oto-Rhino-Laryngology, Head and Neck Surgery 

Prof Dr med. Mathias Pauschinger 

German Society of Cardiology and Cardiovascular Research (DGK) 

Prof Dr med. Monika A. Rieger

German Society for Occupational and Environmental Medicine (DGAUM) 

Dr med. dent. Herbert Rixecker

German Borreliosis Society (DBG) 

Prof Dr rer. nat. Reinhard Wallich

German Society for Immunology (DGI)

Dr Hendrik Wilking 

Robert Koch Institute (RKI)

**Moderator**

Prof Dr med. Ina B. Kopp 

AWMF Institute for Medical Knowledge Management

### 1.4 Methods

This guideline is an updated version of Guideline No. 013-044 “Cutaneous Manifestations of Lyme Borreliosis” Development Stage S1, which was created by an expert committee in 2009. 

The guideline was developed in accordance with the methodological requirements of the Association of the Scientific Medical Societies in Germany (AWMF) for the development and further development of diagnosis and treatment guidelines. It is an S2k guideline in accordance with the AWMF’s three-phase concept. The guideline committee was interdisciplinary (IDA) and the appointed mandate holders of the expert medical societies were informed of the planned update on 20 June 2021.

Uniform terms are used in order to standardise the guideline’s recommendations. The following gradation shall apply:

- **Strong recommendation: “shall” **
- **Recommendation: “should” **
- **Open recommendation: “may be considered” **
- **Recommendation against an intervention: “should not” **
- **Strong recommendation against an intervention: “shall not” **

## 2 The microbiology of the pathogen

**Five of the six human-pathogenic genospecies from the *Borrelia (B.) burgdorferi* sensu lato complex have been isolated in Europe:** *B. afzelii* is the most common, followed by *B. garinii*, *B. bavariensis*, *B. burgdorferi* sensu stricto and *B. spielmanii* [4], [5], [6], [7].

Human pathogenicity has yet to be confirmed for* B. valaisiana*, *B. lusitaniae* and *B. bissettiae*. All of the species that have been confirmed to be human-pathogenic can be found in Europe with the exception of *B. mayonii*; in the USA only *B. burgdorferi* sensu stricto and *B. mayonii* are found, and in Asia all of the species are found with the exception of *B. burgdorferi* sensu stricto and *B. mayonii*. The various genospecies of the *B. burgdorferi* sensu lato complex are genetically very heterogenic [8] and exhibit an organotropism in human infections. Erythema migrans is caused by all 5 genospecies present in Europe, in rare cases possibly also by the relapsing fever *Borrelia B. miyamotoi* [9]. Acrodermatitis chronica atrophicans is almost exclusively caused by *B. afzelii*; *B. garinii* and *B. bavariensis* are often found in conjunction with neurological manifestations, and *B. burgdorferi* sensu stricto mainly affects the joints [7], [10]. *B. spielmanii* has so far only been isolated from erythema migrans [7], [11]. 

## 3 Epidemiology

Lyme borreliosis predominantly occurs between the 40^th^ and 60^th^ parallels of the northern hemisphere. Few relevant epidemiological investigations have been conducted in Europe. A population-based study in southern Sweden found an incidence of 69 per 100,000 inhabitants [12]. In a prospective, population-based study of the region around Würzburg over a 12-month period, 313 cases of Lyme borreliosis were identified, which corresponds to an incidence of 111 per 100,000 inhabitants [13]. In terms of early manifestations, localised erythema migrans was diagnosed in 89% of patients and disseminated erythema migrans in a further 3%. Borrelial lymphocytoma was established in 2% of patients, early-stage neuroborreliosis in 3%, and carditis in <1%. With regard to the late forms of the disease, Lyme arthritis occurred in 5% of patients and acrodermatitis chronica atrophicans in 1%. No chronic neuroborreliosis was identified. 

Currently there is an obligation to report Lyme borreliosis in nine states in Germany (see Annex 4 in Attachment 1) [14]. Epidemiological data obtained through this partial reporting obligation are only based on the clearly diagnosable manifestations, such as erythema migrans, acute neuroborreliosis and acute Lyme arthritis. Thus, it can be assumed that there is a considerable underreporting of cases [14], [15]. A secondary analysis of health insurance data based on the ICD 10 coding A 69.2 (G) found much higher case numbers [16]. A recent study of billing data from statutory health insurances between 2010 and 2019 found that the annual incidence of newly diagnosed Lyme borreliosis cases ranged between 240 per 100,000 insured persons in 2011 and 158 per 100,000 insured persons in 2015 [17]. 

It can therefore be concluded that the incidence of Lyme borreliosis cannot be conclusively established using the current epidemiological data. Data published to date in Germany indicate that the incidence of Lyme borreliosis is somewhere between 60,000 and 200,000 cases per year, or around 72 to 241 cases per 100,000 inhabitants. 

**Figure 1:**
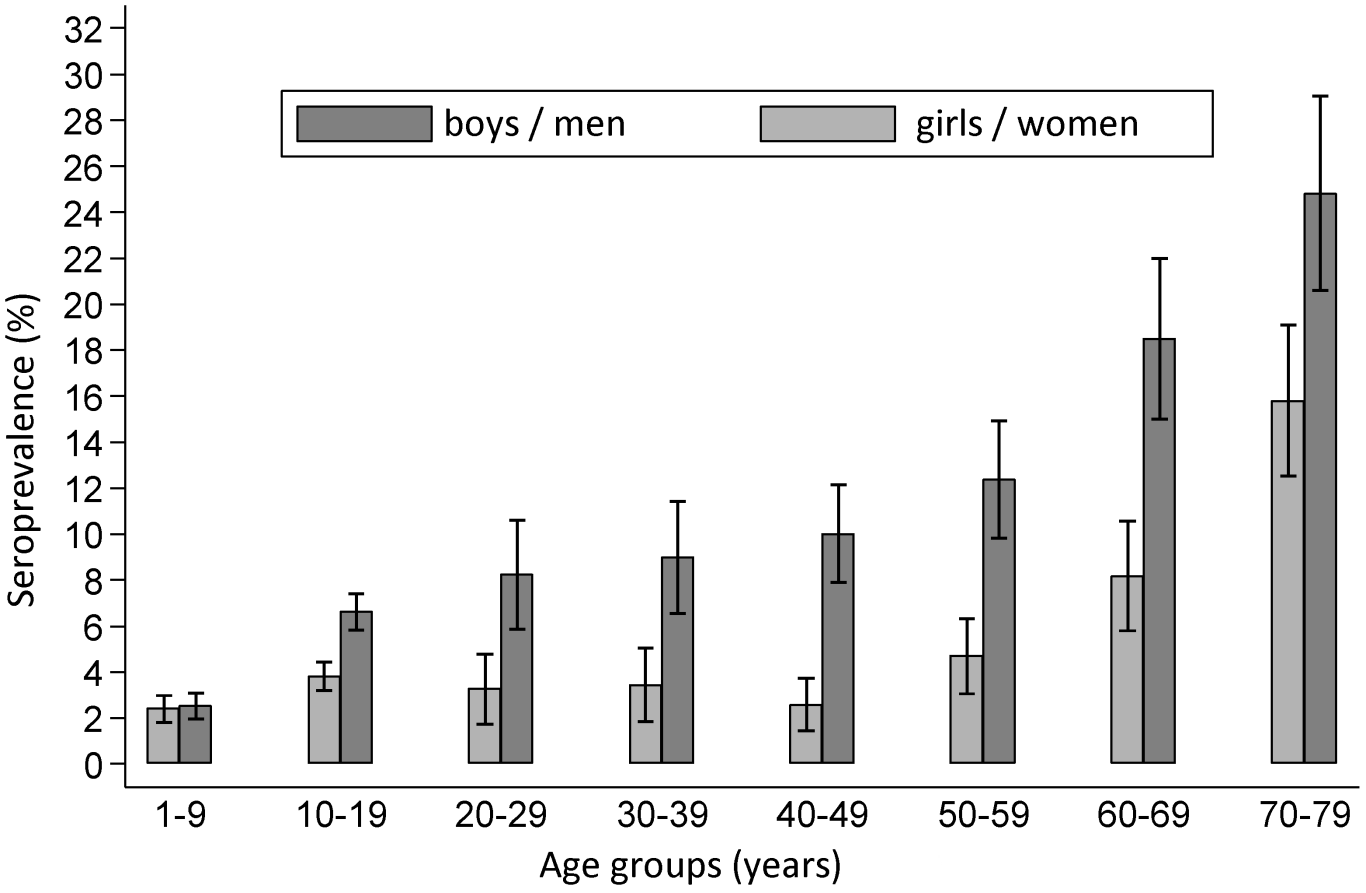
Seroprevalence of *B. burgdorferi* antibodies in Germany – the KIGGS and DEGS studies [18]

A major, nation-wide seroprevalence study of children (KIGGS) and adults (DEGGS) found that the percentage of Borrelia-specific antibodies in serum rises as population age increases. In 14- to 17-year-olds it is as high as 7%, while in adults, the percentage of Borrelia antibodies is even higher. In the cohort of 70- to 79-year-olds, 24.5% of men and 16.4% of women are seropositive (Figure 1 (Fig. 1)) [18]. 

A comparison between two nationally conducted seroprevalence studies (1997–1999 and 2008–2011) shows an annual seroconversion rate of 0.45% (95% CI: 0.37–0.54) of the previously seronegative population. The annual seroreversion rate of the previously seropositive population was 1.47% (95% CI: 1.24–2.17). There was no significant change in seroprevalence between the two study periods [19]. 

A prospective study of the incidence of Lyme borreliosis in southern Sweden and Finland (2008–2009) revealed that a *Borrelia burgdorferi* infection occurred in 78 (5%) of the 1,546 people bitten by a tick. Of these, only 45 (3%) experienced a seroconversion and 33 (2%) developed an infection. Erythema migrans was diagnosed in 28 patients, one person had borrelial lymphocytoma, two people had an acute case of neuroborreliosis, and 2 had non-specified symptoms which were diagnosed as Lyme borreliosis [20]. 

## 4 Transmission routes

*B. burgdorferi* is transmitted to birds, mammals and humans from hard-bodied ticks of the *I. ricinus/I. persulcatus* spp. complex during the blood meal. In Europe it is primarily transmitted from *I. ricinus*, in northeastern Europe and Asia from *I. persulcatus*, and in the USA predominantly from* I. scapularis*.

Often the patient does not recall being bitten by a tick [21]. Ticks suck blood in the course of their cycle of development from larva to nymph to adult tick and before they lay eggs. It is at this time that they can acquire and/or transmit Borrelia. Small rodents – particularly mice – and birds are the primary reservoirs. Birds contribute to the geographical spread of the infected ticks. In Germany, ticks are ubiquitously infected with Borrelia, however percentages can vary greatly from region to region, even between areas very close in proximity (e.g. 4–21%, [22]). 

The successful transmission from tick to mammal is the result of a specific, highly complex vector-pathogen interaction. First the Borrelia are activated in the tick’s gut. Then they migrate to the salivary glands where they bind immunosuppressive salivary proteins to their surface [23]. Finally, they are secreted with the saliva into the bite wound where they are – at least partially – protected from the host’s immune system by immunomodulating substances from the tick’s saliva. This is most likely what allows them to reach a sufficiently high infection dose. A similar transmission through blood-sucking insects is close to impossible due to the short blood sucking time (lack of vector competence in insects for *B. burgdorferi*). Xenobiotic tests reveal that it can take hours for the Borrelia to be transferred depending on the species [24].

See the guideline report for the **dissenting opinion** of the Borreliosis and FSME Alliance of Germany on transmission from mosquitos [25].

When there is an increased occupational risk of a tick bite, cases of Lyme borreliosis (occupational disease 3102, diseases transmitted from animals to humans) should be reported to the accident insurer by the attending physician or employer as an occupational disease in line with Art. 202 of the Social Security Code VII (see Annex 4 in Attachment 1).

## 5 Pathogenesis

The pathogenesis of the borrelial infection is primarily determined by two factors:

1. The pathogen’s evasion strategies [26], [27], [28], [29]
2. The quality of the host’s immune response [30], [31], [32], [33], [34], [35], [36], [37]

Moreover, the salivary proteins released by the tick during the blood meal have also been found to have immunosuppressive effects [38], [39], [40], [41], [42], [43], [44], [45], [46].

Host-specific inflammatory reactions in the skin have also been found to influence the course of the infection [47], [48].

Some of the many strategies the Borrelia use to evade the host’s immune system include the ability to mask their cell surface with proteins/inhibitors from the tick or the host, and to modify their phenotype expression of cell surface proteins (outer surface protein: osp) depending on their environment [49], [50], [51], [52].

Several Borrelia species form a resistance to complement-mediated lysis by binding the regulators of the complement cascade (factor H) to their surface [53], [54], [55], [56], [57]. By binding to plasminogens, Borrelia are capable of breaking down collagen, fibronectin and laminin [56], [58], [59], [60], [61] and disseminating in the skin. 

The innate immune system recognises the Borrelia mainly by their surface proteins (osp lipoproteins) [62], [63], [64], [65], [66]. This interaction leads to the activation of soluble factors, such as the complement system, as well as to the activation of target cells, like macrophages and dendritic cells, and to the induction of inflammatory cytokines [67], [68], [69], [70]. As the infection progresses, specific immune responses are generated, particularly the activation of T helper cells and B lymphocytes, and the production of Borrelia-specific antibodies [28], [71], [72], [73]. In reservoir hosts, like wild mice, the antibodies that form during an infection are able to prevent disease, however they are unable to eliminate the pathogen. In contrast, the antibodies that form in human patients are often unable to prevent disease. Antibodies against certain Borrelia antigens have, however, also been shown to protect against subsequent infection in humans (see vaccines). 

Humans do not acquire permanent immunity following a wild-type infection. As a result, reinfections can occur. 

## 6 Clinical manifestations of Lyme borreliosis

Lyme borreliosis is a multi-organ inflammatory disease. It initially manifests as a localised infection of the skin called erythema migrans. Because of its mild symptoms, this early inflammation of the skin can be overlooked or is not visible. The Borrelia can spread haematogenously. This is clinically recognisable by flu-like symptoms or disseminated erythema on the skin. As the disease progresses, manifestations can appear in other organs, primarily in the nervous system and joints. The disease progresses very differently depending on the individual. Therefore, classifying the disease into stages is not particularly helpful. A distinction between early and late manifestations is preferable since the clinical picture determines both diagnosis and treatment (Table 1 (Tab. 1)). European studies show that Lyme borreliosis manifests as a skin disease in 80–90% of patients and affects other organs in around 10–20% of patients [12], [13], [14], [20].

See the guideline report for the **dissenting opinion** of the Borreliosis and FSME Alliance of Germany on the frequency of erythema migrans [25].

### 6.1 Early localised cutaneous infections

#### 6.1.1 Erythema migrans

**Figure 2:**
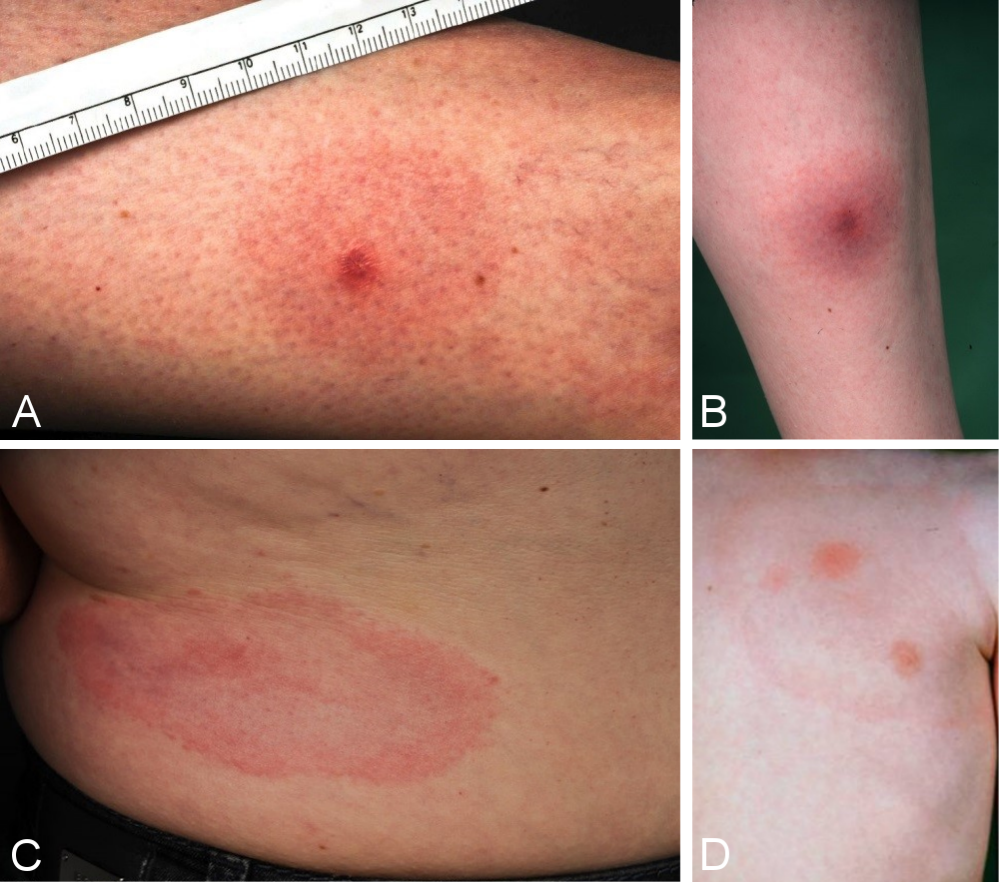
Clinical variations of erythema migrans A: initial seronegative erythema migrans one week after tick bite, DD reaction to insect bite; diameter of 4.9 cm, progressive under observation; B: seronegative erythema migrans, increase in IgM antibodies 5 days after start of treatment; C: typical marginated, migrating erythema migrans, D: typical marginated erythema migrans with fresh areas of inflammation inside the ring

A localised skin infection can occur in the area around the infecting tick bite anywhere from 3 to 30 days following the tick bite [74]. The extent and duration of the inflammatory reaction varies greatly between individuals. A diagnosis of erythema migrans requires the diameter of the erythema to be more than 5 cm (Figure 2A and B (Fig. 2)) [75].

A clinically clear presentation of a typical **erythema migrans** is a marginated erythema – not raised, not overheated – with centrifugal dissemination around the tick bite (Figure 2C and D (Fig. 2)).

**Features of a typical solitary erythema migrans**

- Time interval between tick bite and onset of the erythema is typically 3 days to several weeks
- Increasing centrifugal spread of the erythema (crescendo reaction)
- Marginated, non-raised erythema that is at least 5 cm in diameter
- Visible tick bite site at the centre of the erythema
  (Strong consensus 13/0/0)

#### 6.1.2 Variability of erythema migrans (atypical erythema migrans)

**Figure 3:**
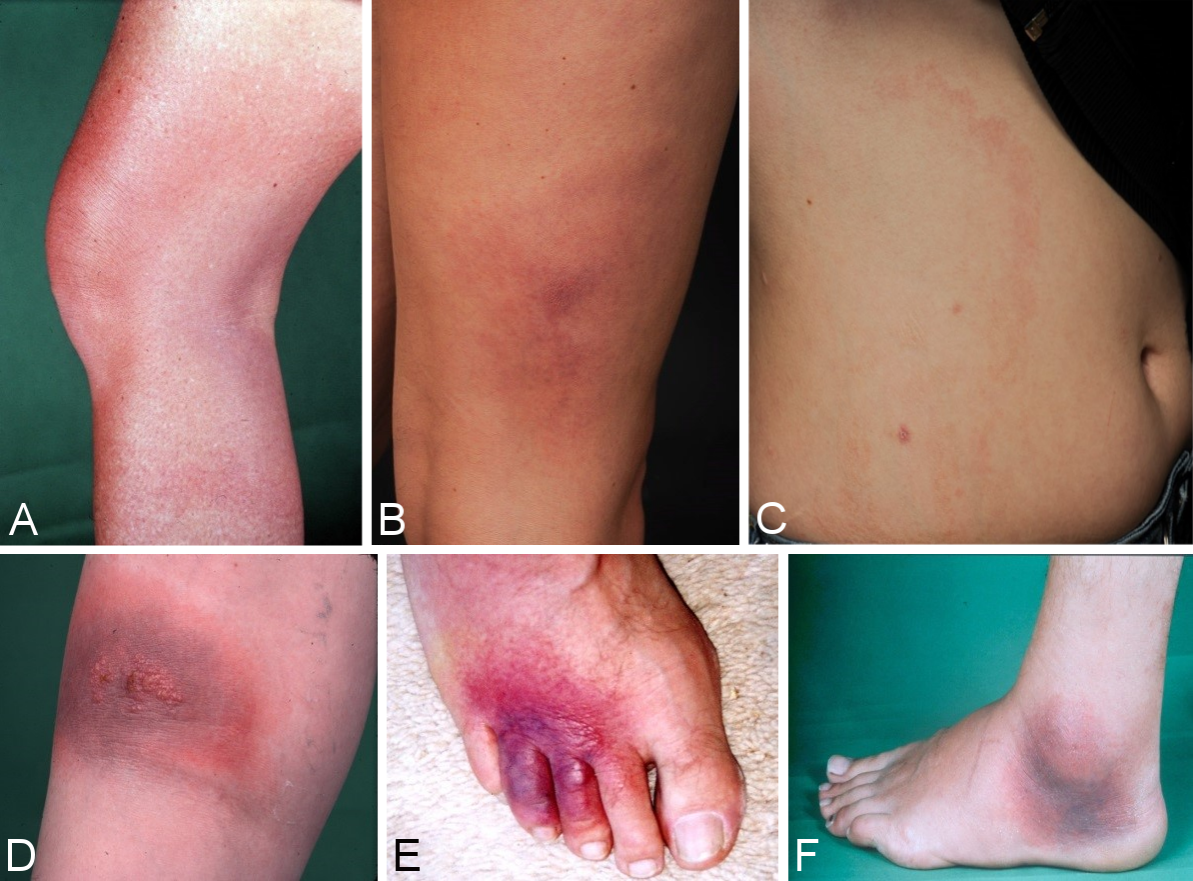
Variability of the erythema migrans A: flaming red erythema chronicum migrans with radiculitis on the left leg, DD erysipelas; B: blotchy purple erythema chronicum migrans persisting on the upper thigh for 3 months; C: Large light-red arch-shaped erythema chronicum migrans on the abdomen; D: centrally vesicular erythema migrans; E: Haemorrhagic bullous erythema migrans on the foot; F: purple, haemorrhagic, non-migrating erythema chronicum migrans on the outer ankle with joint swelling

The initial skin infection may be clinically ambiguous. Borrelia have been detected in homogenously red and non-migrating erythema, in patchy and infiltrated erythema (Figure 3B (Fig. 3)), in erysipelas-like flaming red erythema (Figure 3A (Fig. 3)) and in centrally vesicular erythema (Figure 3D (Fig. 3)) [76], [77]. The inflammation can completely disappear in the middle and fade to such as extent that the erythema is only visible around the borders – where the Borrelia are migrating – when heat is applied (Figure 3C (Fig. 3)). The erythema can also be haemorrhagic, particularly on the lower extremities (Figure 3E and F (Fig. 3)). The centre can turn dark purple in colour (Figure 3F (Fig. 3)). The border can be raised or urticarial. The original site of the tick bite is identifiable in the centre as a red papule (Figure 2A and B (Fig. 2)) [76], [78]. In the case of multiple erythema migrantia, erythema also develops at sites other than the site of the bite. **Without antibiotic treatment** the Borrelia can persist for months or years in the skin and the erythema can slowly spread over the body. Often the red border is the only evidence of the inflammatory reaction to the migrating Borrelia. If the erythema migrans persists for several weeks to months, it is referred to as **erythema chronicum migrans** [79]: >4 weeks. In most cases (approx. 80%) serological detection of the IgG antibodies (sometimes even the IgM antibodies) is possible [80].

The erythema can subside even without antibiotic treatment. Spontaneous healing is possible, however the Borrelia can persist even without a visible inflammatory reaction, and, after a period of latency, this can lead to manifestations in other organs. 

**Variability of the erythema migrans (atypical erythema migrans)**

- Non-migrating
- Not marginated
- Infiltrated instead of macular
- Centrally vesicular
- Haemorrhagic
- Irregular patches
- Only visible when skin is warmed
- No visible tick bite site
  (Strong consensus 13/0/0)

**Concluding recommendation:**

Due to the extraordinary variability of the clinical presentation, atypical erythema migrans is difficult for dermatologically inexperienced physicians to diagnose. Therefore, patients with atypical erythema should be referred to a dermatologist. (Strong consensus 13/0/0)

#### 6.1.3 Borrelial lymphocytoma

**Figure 4:**
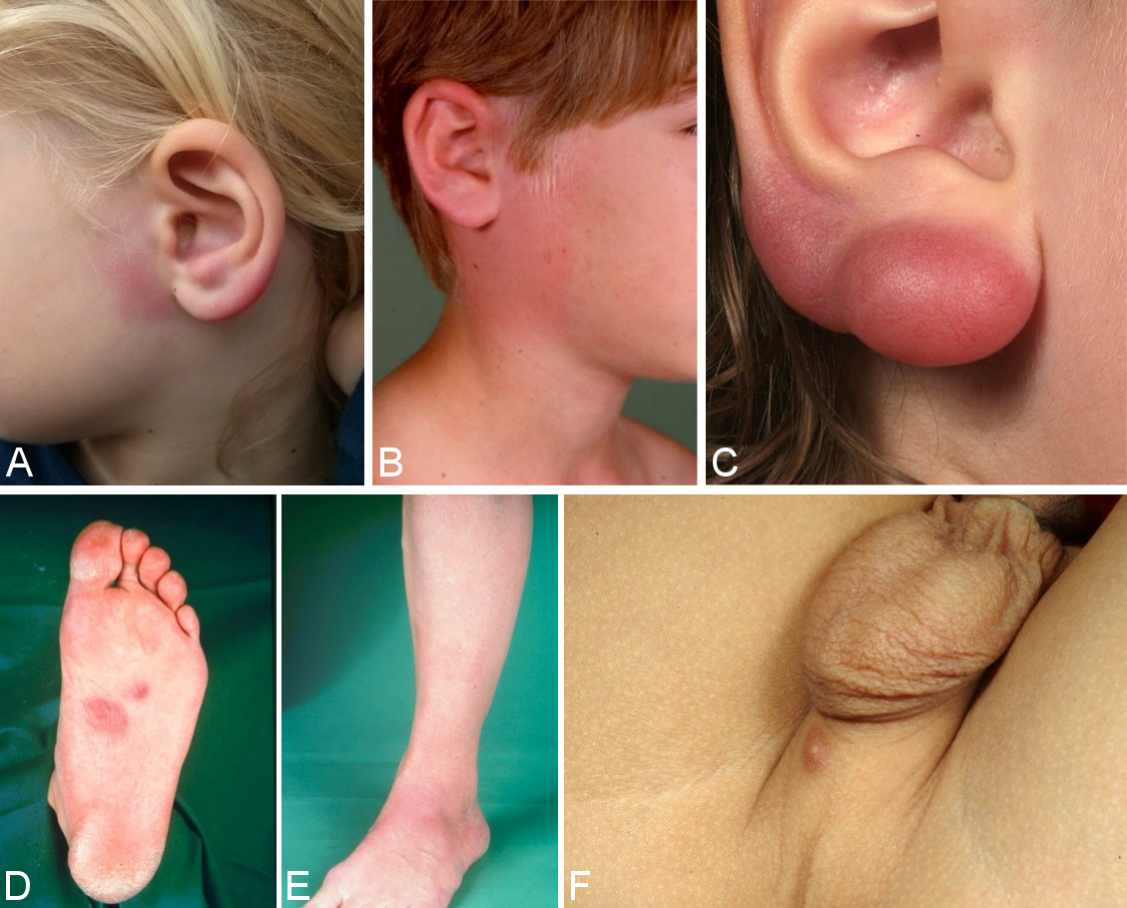
Borrelial lymphocytoma A: Borrelial lymphocytoma preauricular and on the left earlobe; B: Borrelial lymphocytoma on the right auricle near a non-marginated erythema migrans; C: pronounced knotty borrelial lymphocytoma on the earlobe; D, E: Borrelial lymphocytoma on the sole of the foot with erythema migrans on the lower leg, initially misdiagnosed histologically as a low-grade B cell lymphoma; F: small perineal borrelial lymphocytoma

In the early stages of the disease, pseudolymphoma (cutaneous lymphoid hyperplasia) (Figure 4B (Fig. 4)) can occur at the site of the tick bite or in the migrating erythema migrans. In most cases it is solitary; in rare cases it is also disseminated. Borrelial lymphocytoma occurs more frequently in children than in adults (in 7% of children and in only 2% of adults with Lyme borreliosis [12]). The preferred sites in children are the earlobes (Figure 4A and C (Fig. 4)), nipples and genitoanal area (Figure 4F (Fig. 4)) [81], [82]. The disease was first described as lymphadenosis cutis benigna by Bäferstedt in 1944. *B. burgdorferi* s.l. is detectable in the pseudolymphomas [83]. In most cases it is *B. afzelii* [84]. Histologically, there are mixed B and T lymphocytic infiltrates. However purely B cell infiltrates can also occur, which are difficult to differentiate from low-grade B cell lymphoma (Figure 4D and E (Fig. 4)) [76]. Borrelial lymphocytoma can also occur in the late stages of the disease within the context of acrodermatitis chronic atrophicans [81]. 

In the case of borrelial lymphocytoma, there is a substantial increase in the number of IgG antibodies in the serum regardless of the duration of the infection [85], [86]. In rare cases, multiple borrelial lymphocytomas can occur in the early disseminated stage or even in the late stages of the disease. Here accurate, histological, immune-histochemical and molecular-genetic clarification is required so that a differential diagnosis can be made from malignant cutaneous lymphomas.

**Important features of a borrelial lymphocytoma**

- Pseudolymphoma, mostly solitary, more frequent in children
- Localised, primarily on the earlobes, nipples or in the genital area
- Purple subcutaneous nodules or plaque
- Histologically, mostly mixed B and T lymphocytic infiltrates
  (Strong consensus: 13/0/0)

### 6.2 Early disseminated cutaneous manifestations 

Some patients experience haematogenous dissemination in the early stages of the disease, which is clinically identifiable by flu-like symptoms such as a slight fever, arthralgia, myalgia, headache, lymphadenopathy and multiple erythemata migrantia. This stage is very difficult to diagnose if no erythemas are visible or if they cannot be identified because they have an atypical morphology. 

#### Multiple erythemata migrantia (MEM) 

**Figure 5:**
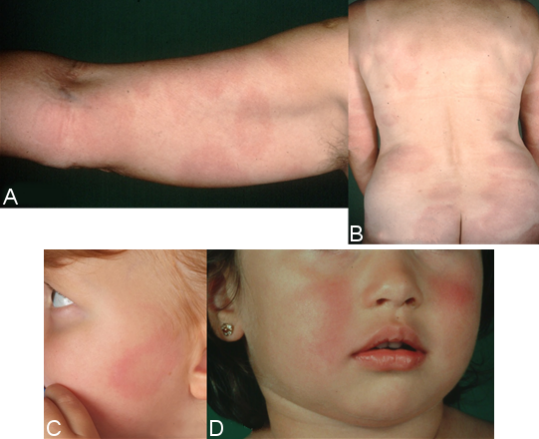
Multiple erythemata migrantia (MEM) A, B: pronounced erythemata migrantia on the right arm, approx. 40 more on the torso and lower extremities; C: oval erythema on the left cheek, several similar erythema on the torso and thigh during early disseminated borreliosis; D: symmetrical redness on the cheeks in a 5-year-old girl with multiple erythemata migrantia on her torso and extremities, accompanied by flu-like symptoms

The haematogenous dissemination of the Borrelia in the skin is identifiable by the many sharply defined, symptomless, oval erythemas of varying sizes, i.e., multiple erythemata migrantia (Figure 5B and C (Fig. 5)) [77], [87], [88]. Children often experience symmetrical erythema on their face, similar to fifth disease (Figure 5A (Fig. 5)) [76], [77]. MEM can be associated with systemic symptoms and acute neurological symptoms [89]. The histological picture is initially atypical. The typical perivascular plasma cell infiltrates can only be found in the advanced stage of the disease. There is usually a highly elevated number of IgM antibodies in the serum, or the antibodies increase rapidly once treatment begins. There is usually an elevated number of IgG antibodies. Borrelia taken from skin lesions, and, in rare cases, blood can be cultivated, or their DNA can be detected by PCR [88], [90]. 

Important features of multiple erythemata migrantia

- Symptomless, disseminated round or ovular redness on the skin (strong consensus: 13/0/0)
  - No epidermal changes
  - Ring-shaped or homogenous
  - In children, often symmetrical erythema on the face (similar to fifth disease)
  - Persisting days or weeks
  - Recurring at the same sites
  - Possibly associated with systemic or acute neurological symptoms
    (Strong consensus: 13/0/0)

### 6.3 Late cutaneous manifestations 

#### Acrodermatitis chronica atrophicans (ACA) 

The disease can manifest in various organs after varying periods of time – from months to years – depending on the individual. Chronic skin infections mostly occur in older patients and more frequently in women [3], [91]. 

A large retrospective descriptive cohort study conducted over 28 years (1991–2018) of 693 Slovenian patients who presented with ACA has now been published for the first time. Clinical and microbiological findings were anal-ysed. A diagnosis was made after a mean time of 12 months following the onset of changes to the skin. The lower extremities were affected in 70% of cases. In 55% of patients, the ACA was localised to one extremity, in 31.3% to 2, in 5.6% to 3 and in 7.8% to all 4 extremities. Bilateral involvement was observed in 42.1% of cases. Fibroid nodules were also reported in 2.2% of patients, concomitant arthritis in 2.6% and in 20.8% of patients, the ACA was associated with peripheral neuropathy. The neuropathic symptoms only occurred in the extremity affected by the ACA [3].

Isolated cases have also been reported in children [86], [92], [93], [94], [95].

#### Oedematous-infiltrative stage of ACA 

**Figure 6:**
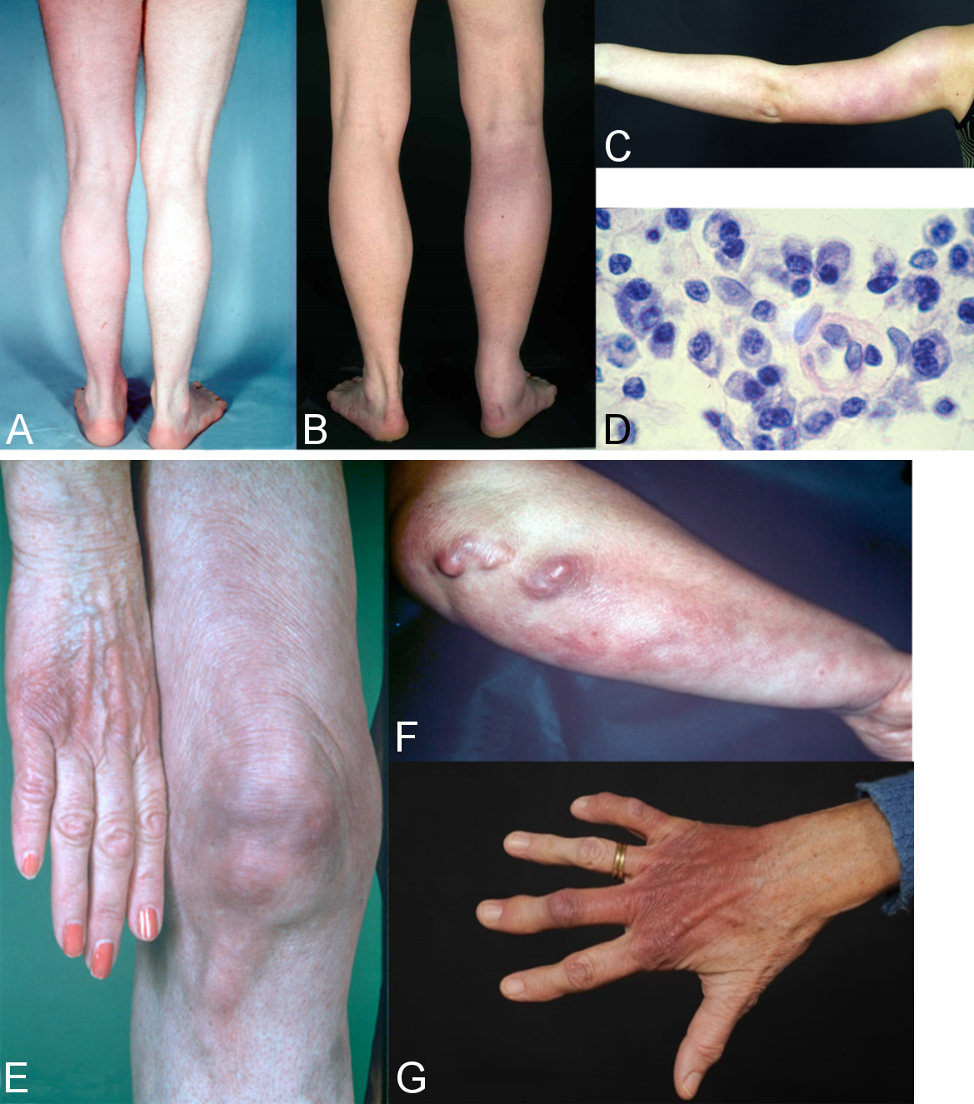
Late cutaneous manifestations A–D: Oedematous-infiltrative stage of acrodermatitis chronica. A: Acrodermatitis chronica. Homogenous reddening of the left leg without atrophy, persisting for one year; B Acrodermatitis chronica in the oedematous-infiltrative stage. Pronounced swelling and purple discolouration of the right leg with nodular enlargement of the Achilles tendon and swelling of the ankle joint; C: Acrodermatitis in the oedematous-infiltrative stage. Blotchy purple confluent erythema on the left arm of a 15-year-old girl; D: typical perivascular plasmacellular infiltrate in acrodermatitis chronica E–G: Atrophic stage of ACA. E: ACA Purple discolouration and atrophy on the back of the right hand and little finger, and purple blotches, bands and infiltrates dorsally on the right knee; F: ACA with ulnar stripes and purple blotches on the right underarm and pronounced purple fibroid nodules below the elbow; G: ACA dark red to purple discolouration and atrophy of the right hand dorsally (so-called “baked apple skin”) with swelling of the finger joints

Acrodermatitis initially manifests as pink reticular, then increasingly purple, oedematously infiltrated cushion-like erythema, usually on the extremities. The skin is excessively warm, however there is initially no pain apart from a feeling of heaviness. This is the oedematous-infiltrative stage of acrodermatitis chronica (Figure 6A and B (Fig. 6)). These purple infiltrates can also appear on the face and be mistaken for lupus erythematosus or cutaneous malignant lymphoma [76].

#### Atrophic stage of ACA 

In the course of the disease there is increasing atrophy of all skin layers and skin appendages. Occasionally coarse, juxta-articular fibroid nodules and band-shape stripes appear (Figure 6F (Fig. 6)), e.g. rare but typical inflammatory ulnar stripes and swelling in the heel and Achilles tendon, or in other joints around the ACA, even in the oedematous-infiltrative stage (Figure 6B (Fig. 6)). As the disease progresses, circumscribed fibrosis or pseudoscleroderma develops near the ACA, which can be mistaken for circumscribed scleroderma. Arthritis, arthralgia and myalgia in the affected extremities are frequently associated with ACA [91]. 

A peripheral neuropathy occurs in 40–60% of patients in connection with ACA, which is characterized by a feeling of numbness, a tingling sensation, burning and an increased sensitivity to pain (allodynia) [96], [97], [98]. Without antibiotic treatment, Borrelia can live for years in the skin and in the fibroid nodules [99]. 

As the disease progresses, all of the affected skin atrophies and there is a loss of body hair as well as connective and fatty tissue (Figure 6E and G (Fig. 6)). 

When the changes to the ACA-affected skin are symmetrical, they are difficult to differentiate clinically from age-related skin atrophy, acrocyanosis and chronic venous insufficiency. Histologically, acrodermatitis chronica atrophicans is characterised by a pronounced perivascular plasma cell-rich inflammatory infiltrate throughout all layers of the skin (Figure 6D (Fig. 6)) and, in the late stages, by an increasing atrophy of the epidermis, connective tissues and fatty tissues [100].

An ACA diagnosis is based on a typical clinical presentation, a typical histology and, as a rule, a strong positive detection of Borrelia IgG antibodies in serum [3], [91]. In ambiguous cases, particularly in the case of borderline antibody concentrations, a diagnosis must be confirmed by histological analysis and DNA detection, or by cultivating Borrelia from the skin. 

**Important clinical features of acrodermatitis chronica atrophicans (ACA)**

- Initial oedematous-infiltrative stage (plasmacellular dermatitis), reddish discolouration of the skin, usually on one extremity
- As the disease progresses, transition to the atrophic stage: purple to loam-brown discolouration of the skin, skin atrophy, loss of body hair, loss of connective and fatty tissues, protrusion of blood vessels, juxta-articular fibroid nodules and joint involvement
- Association with a peripheral neuropathy in around 50% of cases
- Older women more likely to be affected
  (Strong consensus: 13/0/0)

### 6.4 Manifestations in the nervous system and joints associated with cutaneous borreliosis 

Early borreliosis with erythema migrans can be accompanied by early neuroborreliosis. Arnez et al. found pleocytosis in the cerebrospinal fluid in 26% of the 214 children with multilocular erythemata migrantia that they studied. Of these, 11% had clinically symptomatic lymphocytic meningitis [101]. Bannwarth Syndrome (radiculoneuritis) with characteristic nocturnal pain can also occur – in rare cases with paresis of the cranial or peripheral nerves. 

A peripheral neuropathy of the affected extremity occurs in 50% of patients with ACA [97].

Rheumatological symptoms, especially myalgia and arthralgia, can occur relatively early on in the disease alongside erythema migrans. Attention should be paid to cardiac symptoms with dysrhythmia (typically AV block of varying degrees), which can occur in conjunction with or following a case of erythema migrans. 

Frequently, the joint adjacent to the erythema migrans is affected. This typically manifests as acute intermittent arthritis of the large joints with voluminous, at times painful joint swelling, usually in the form of mono- or oligoarthritis. Knee joints are affected in 85% of patients. The, often, extensive knee joint effusions lead unusually frequently and early on to the development of popliteal cysts (Baker’s cysts). Ankle and elbow joints are less frequently affected and no involvement of the finger joints, especially in the form of polyarthritis, has been observed. Lyme arthritis is usually episodic, i.e. with recurring bouts of inflammation that alternates between periods of light to no symptoms. 

### 6.5 Primary cutaneous lymphomas with a B. burgdorferi infection 

A recent meta-analysis on the association between *B. burgdorferi* and primary cutaneous lymphomas found there is a significant association in areas where Borrelia are endemic. Here no differences were detectable between the different lymphoma entities (both B-cell and T-cell lymphomas). The authors recommend molecular testing for *B. burgdorferi* and, if necessary, antibiotic treatment for patients from endemic areas that have primary cutaneous lymphoma. However, further studies are deemed necessary to confirm this finding [102]. 

### 6.6 Differential diagnoses for cutaneous Lyme borreliosis

The most common differential diagnoses for cutaneous Lyme borreliosis are listed in Table 2 (Tab. 2). 

**The multitude of differential diagnoses are evidence that, with the exception of typical erythema migrans, most cutaneous manifestations of Lyme borreliosis require careful dermatological testing.** In particular, a poor response to antibiotic treatment should not be uncritically interpreted as persistent borreliosis that is then treated for months with antibiotics. In such cases, the initial diagnosis must be critically questioned and a differential diagnosis be made. This avoids unnecessary, potentially harmful antibiotic treatment or a delay in treatment for other diseases, or it confirms the initial diagnosis. 

It is therefore recommended that patients whose ambiguous skin afflictions persist after treatment, be referred to dermatologists or to paediatricians with dermatological experience. 

**Recommendation**

Skin inflammations that were diagnosed as Lyme borreliosis and which have not healed after *lege artis* antibiotic treatment shall be referred to a dermatologist. (Strong consensus 13/0/0)

## 7 Diagnostic testing

### 7.1 Indirect pathogen detection (serodiagnosis, antibody detection) 

Due to the complex characteristics of the pathogen, indirect pathogen detection using serological methods continues to play a key role in the diagnosis of Lyme borreliosis in routine laboratory medical care. In accordance with the methods and standards required in Germany, antibodies are detected in serum using a **step-by-step diagnostic approach** made up of a standardised **screening test** (immunoassay: ELISA, CLIA etc.) and a **confirmation test** (immunoblot). This ensures that the diagnostic procedure achieves the highest possible sensitivity and specificity (Table 3 (Tab. 3)). 

In Europe, serology tests for Borrelia are not subject to any form of mandatory, extensive or independent clinical evaluation as part of the approval process. This means that there is a range of commercially available test formats. In addition to various types of immunoassays, there are also a variety of test antigen preparations that use native and recombinant antigen combinations with, at times, varying performance data [16], [103], [104]. Even though the principles of testing procedures and the interpretation of serology test results are laid down in binding standards in Germany (DIN 2005), it should be noted that the variability and insufficient standardisation of commercial test systems means that the interpretation of test results and, in particular the evaluation criteria for immunoblot tests, vary among manufacturers and must be carried out according to the respective manufacturer’s specifications. Meta-analyses conducted as part of external quality controls highlight the ongoing issue of insufficient standardisation of testing and comparability of results [16], [105]. Thus, attending physicians should be aware of the credentials of their diagnostic laboratory as well as the diagnostic assays and test specifications that it uses and, where possible, have the laboratory that performed the initial test perform the follow-up test or, preferably, use the same test while applying a parallel approach with the pre-serum. 

#### Progression of the immune response and the interpretation of findings 

In the course of a natural infection, specific **IgM antibodies** are usually detectable 3–6 weeks after the onset of illness; **IgG antibody titres** peak more slowly (after weeks to months). 

It should be noted that seroconversion may not occur even after timely and **successful** treatment of early manifestations. Furthermore, when IgM antibodies are detected, the immune response may not progress normally in the sense of a conversion from IgM to IgG. In contrast to immune responses to other infectious bacterial diseases, the antibody response with Lyme borreliosis often regresses very slowly after both a latent and resolved infection as well as after successful treatment. As a result, IgM reactivities or specific IgG values may remain detectable for months, even years, after the patient has successfully recovered from the infection. Weakly positive borrelial-specific antibody levels are often a sign of a previous infection in the sense that **antibodies are left over from a past infection (serological scar)** [19], [103], [104], [106]. Nevertheless, a reinfection cannot be ruled out based on these laboratory results. Such results were detected in 20% of people belonging to frequently exposed population groups, e.g. forestry workers, who were examined as part of health screenings without any current or past symptoms of disease [107], [108]. Possible co-incidences of these types of titres, with persisting antibodies from previous infections and unspecified findings, are also possible among the normal population [18], [109] and can be responsible for erroneous interpretations and diagnoses. It is therefore advisable to always complete the ‘Symptoms’ section on the laboratory order. This supports the laboratory in better interpreting the results.

**In immunocompetent patients, the isolated positive detection of IgM antibodies effectively rules out a late manifestation of Lyme borreliosis.**

The use of very sensitive early-phase antigens, such as VlsE, in diagnostic testing means that a specific IgG response can be detected very early on in the course of the infection. As a result, specific IgM antibody results are playing an increasingly limited role in the diagnosis of Lyme borreliosis, especially as IgM detection has a poorer overall specificity than IgG detection [110], [111]. However, positive IgG levels can also persist, sometimes in high concentrations, over a long period of time [19], so that no conclusions can be drawn regarding the activity of Lyme borreliosis or even the need for treatment in the absence of a traditional activity marker, without additional clinical information and only on the basis of positive serological findings. At the same time, a statement can only be made about the significance of changes in the findings if comparison tests are carried out on serum samples that were taken at different times, ideally using a parallel approach with the pre-serum, but, in any case, using the same test [103], [112].

An immunoblot analysis is generally used within the framework of a step-by-step diagnostic approach to confirm the results of the screening test as well as to classify the immune response into an early or late phase based on the band pattern, especially in the case of the IgG immunoblot. Thus, **a narrow spectrum of detected bands with antibodies against early-phase antigens (e.g. VlsE, OspC, p41)** typically corresponds to an early manifestation (e.g. erythema migrans, facial paresis) or a brief latent infection; it does not correspond to an infection that has persisted for some time [103], [106], [112], [113], [114]. In contrast, **a wide band spectrum, including reactions to late-phase antigens (e.g. p100, p17/p18)**, corresponds well to a **late manifestation** (e.g. arthritis, acrodermatitis) [106], [113], [114] as well as to the asymptomatic persistence of antibodies (serological scar). It primarily does not, however, correspond to an early manifestation or a short duration of the disease. **Reinfections** are difficult to detect based solely on serology test results and without additional clinical information. They are only detectable by a clear **increase in IgG levels or significant changes in the immunoblot band pattern** in serum samples examined in parallel. 

The immunoblot must be interpreted by an experienced laboratory technician, making it time-consuming and expensive, and results can vary depending on the examiner. Therefore, it has been suggested that the immunoblot be replaced by another ELISA – e.g. with C6 peptide [115], [116]. This simplifies the serology test. As the result depends on the quality of the primary ELISA, both parts of the two-step test procedure can be carried out consecutively or in parallel [115], [117]. In the USA, several ELISAs have already been authorised by the FDA to be used in such a procedure. However, this can naturally lead to a loss in specificity. This procedure also makes it impossible to assess the development of the immune response – in the sense of a widening of the band pattern – which can be important for the serological assessment of late or chronic forms of the disease. Further investigations need to be conducted to establish whether this approach is also suitable for Europe, and if so to what extent. 

**One major premise of serological testing for Lyme borreliosis is that the requesting physician is aware that these types of tests should only be ordered if there is reasonable clinical suspicion, i.e. the patient’s clinical findings and subjective complaints must always be taken into account. **Only when there is sufficiently high pre-test probability (prevalence of Lyme borreliosis in the patient cohort being investigated >20%) can it be assumed that there is a sufficiently utilisable positive predictive value of a positive test result [118]. If the test is only ordered to rule out Lyme borreliosis when there are unspecified or non-typical disease symptoms, the positive predictive value of the laboratory test drops to almost zero with respect to the possible confirmation of Lyme borreliosis. However, due to the relatively low overall prevalence and incidence of Lyme borreliosis among the general population, a negative test result has an excellent negative predictive value for ruling out the disease in immunocompetent patients with persisting symptoms. 

**Recommendations**

- Serology testing shall only be ordered when there is sufficient clinical suspicion. (Strong consensus: 13/0/0)
- Testing shall be performed using a step-by-step diagnostic approach (screening and confirmation test). (Consensus: 11/2/0)
- A positive antibody test is not proof that Lyme borreliosis is clinically present. (Strong consensus: 13/0/0)
- A negative antibody test largely rules out Lyme borreliosis in immune-healthy patients with a protracted length of illness. (Consensus: 11/2/0)
- An isolated positive IgM test does not support a late manifestation of Lyme borreliosis. (Consensus: 11/2/0)

See the guideline report for the **dissenting opinion** of the Borreliosis and FSME Alliance of Germany on assessing serology testing [25].

### 7.2 Direct pathogen detection 

For Lyme borreliosis, the relevant quality standards for microbiological analysis apply to direct pathogen detection using culture and PCR/NAT [106], [119].

#### 7.2.1 Culture 

In the past, direct detection by culture using the modified Barbour-Stoenner-Kelly medium was the gold standard and provided clear proof of an infection with *B. burgdorferi* [120], [121]. Now, however, it is rarely used. Direct detection of skin manifestations by culture are often successful. Detection by culture can be done to a limited degree from cerebrospinal fluid, and very rarely from synovial fluid, synovial biopsies and blood. In individual cases, the detection of *B. burgdorferi* has also been achieved in other tissue samples, e.g. heart muscle and iris [122], [123]. Cultivation from patient samples using suitable media is material- and labour-intensive and commonly takes more than two weeks. European studies have found the sensitivity of these methods to be between 40% and 90% for erythema migrans and between 20% and 60% for ACA [124], [125], [126], [127]. Overview in: [128]. Because of the invasiveness of the sample taking, direct detection by culture should therefore be based on a clear indication and be explicitly restricted to specially designated reference laboratories, such as the National Reference Centre for Borrelia at the Bavarian Health and Food Safety Authority in Oberschleissheim. This is particularly important as further molecular confirmation tests are required in the event of a positive result. 

**Recommendations for direct detection by culture**

- Direct detection by culture should only be performed if the differential diagnosis is inconclusive. (Strong consensus: 13/0/0)
- The cultivation of *Borrelia burgdorferi* sensu lato should remain restricted to specialist laboratories. (Strong consensus: 13/0/0)
- Positive culture results are to be confirmed using suitable molecular methods. (Strong consensus: 13/0/0)

#### 7.2.2 Direct detection using molecular methods 

The detection methods currently being used in Lyme borreliosis testing are regarded as having a low level of standardisation [129]. This applies to DNA isolation from suitable clinical materials, as well as to reaction conditions and the selection of primers. In principle, the detection of Borrelia from a skin biopsy using nucleic acid amplification techniques (NAT, usually PCR) is very reliable and, in the case of early manifestations, is more sensitive than serological antibody detection. The diagnostic sensitivity of NAT for detecting erythema migrans and acrodermatitis chronica atrophicans from biopsies is around 70% [127], [130], [131], [132], [133]. However, molecular confirmation tests (probe hybridisation, sequencing of the amplificate) must confirm the specificity of the positive results and these findings must be reported. 

After treatment, Borrelia DNA can still be detected for weeks – or even months – in the affected patch of skin before conclusions can be drawn as to whether the therapy has failed [134], [135], [136]. The molecular detection of pathogens without the simultaneous presence of typical disease manifestations is not clinically relevant. Direct molecular detection in urine samples is not currently recommended as the diagnostic sensitivity and specificity are unclear [133], [137], [138]. Direct detection by PCR should therefore be based on a clear indication (e.g. skin manifestations with inconclusive differential diagnosis) due to the invasiveness of the sample collection and lack of standardisation. Thus, it should be explicitly restricted to designated specialist and reference laboratories, such as the National Reference Centre for Borrelia at the Bavarian Health and Food Safety Authority in Oberschleissheim. This is particularly important since molecular confirmation tests are required in the event of a positive result. 

**Recommendations for molecular detection**

- Direct molecular detection (PCR) is not a screening test if Lyme borreliosis is suspected. (Strong consensus: 13/0/0)
- A negative PCR test does not rule out Lyme borreliosis. (Strong consensus: 13/0/0)
- A positive PCR test shall be confirmed by further molecular testing methods and the detected genospecies shall be reported in the findings. (Strong consensus: 13/0/0)
- A positive PCR test following treatment with antibiotics in accordance with the guidelines or without a clinically typical manifestation is not an indication for (renewed) treatment with antibiotics. (Consensus: 11/2/0)
- Direct molecular detection should only be used for ambiguous skin manifestations and be conducted in specially designated microbiology labs. (Strong consensus: 13/0/0)

### 7.3 Diagnostic testing of clinical skin manifestations 

#### 7.3.1 Erythema migrans (typical) 

**Figure 7:**
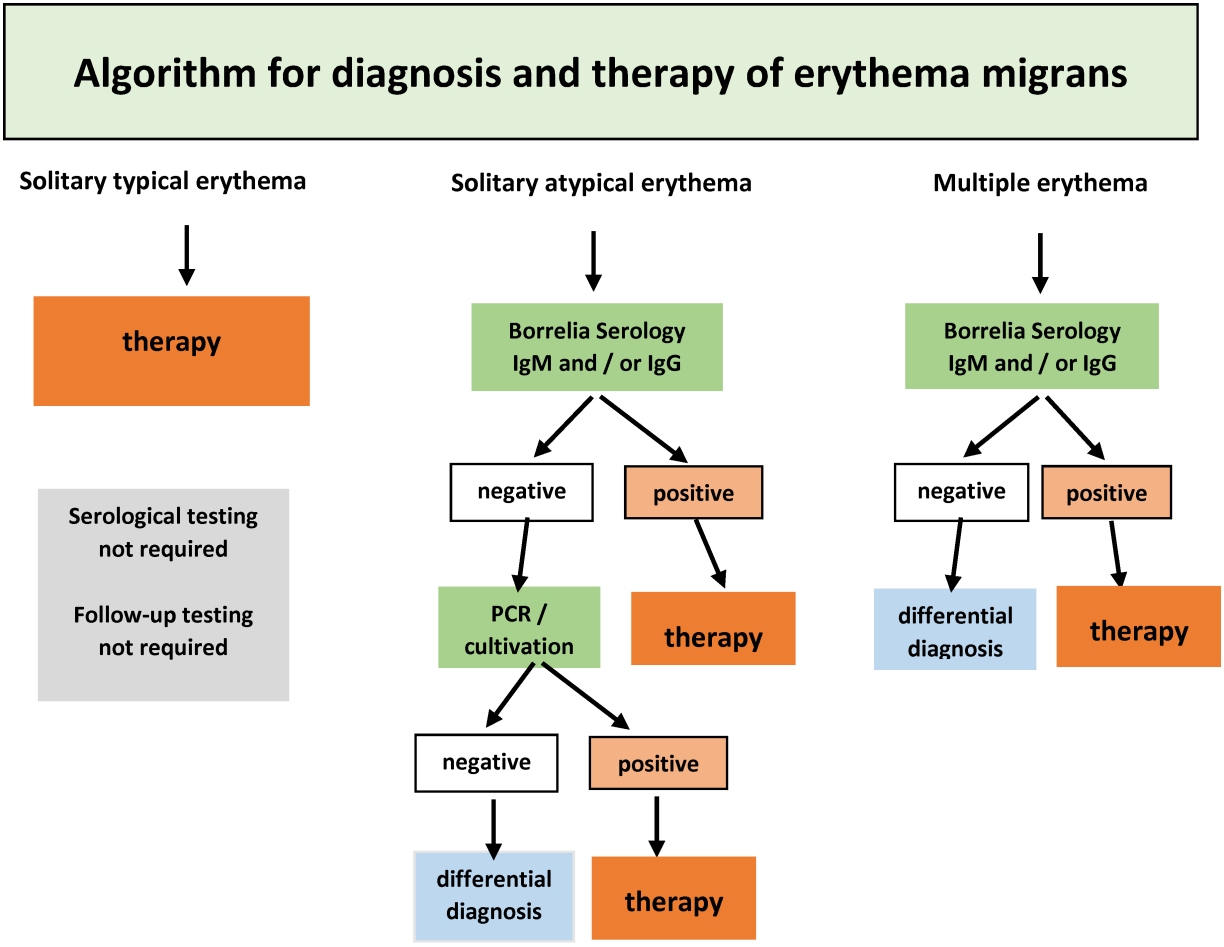
Algorithm for diagnosing a solitary or multilocular erythema migrans

If a **clinically typical erythema migrans** is present (see section on clinical manifestations), no further laboratory testing should be carried out and antibiotic treatment should start immediately (Figure 7 (Fig. 7)).

**Recommendations**

- If a typical erythema migrans is present (see section on clinical manifestations), no further laboratory testing (serological, cultural or molecular) is required. (Strong consensus: 13/0/0)
- If a typical erythema migrans is present, antibiotic treatment shall begin immediately. (Strong consensus: 13/0/0)

#### 7.3.2 Erythema migrans (atypical) 

If an **atypical erythema migrans** is suspected, a serology test shall be carried out in every case (Table 4 (Tab. 4)). If the findings remain ambiguous, pathogen detection should be done using PCR, if necessary, also by culture (Figure 7 (Fig. 7)). A skin biopsy should be taken near the inflamed border. After informing the patient and obtaining written consent, the selected area of the skin is numbed using local anaesthesia. After thorough disinfection of the skin, a 4 mm punch is used to remove the skin. This is then placed in a sterile vessel with a 0.9% saline solution. Direct inoculation in the culture medium only makes sense if the sample can be processed in a laboratory within a few hours, as otherwise, fast growing skin bacteria can make it difficult to cultivate the Borrelia. 

A histological analysis is rarely able to indicate if it is erythema migrans; it can, however, be useful in making a differential diagnosis. 

**Recommendations**

- In the case of an atypical clinical appearance of erythema migrans, suspicion shall be clarified by a serology test. (Consensus: 11/2/0)
- If the serology test is negative and clinical suspicion remains, direct molecular detection or direct detection by culture from biopsy material shall be used for clarification. (Strong consensus: 13/0/0)

#### 7.3.3 Multiple erythemata migrantia (MEM) 

If multiple erythemata migrantia, also known as multilocular erythema migrans **(MEM)** is suspected, serological antibody detection and pathogen detection by PCR and culture from a skin biopsy can be used. A serology test should always be carried out. If the findings remain ambiguous, a PCR test should be used to detect the pathogen, if necessary, also by culture (Figure 7 (Fig. 7)) (see 3.2.1). In the case of MEM, special attention should be paid to clinical signs of extracutaneous symptoms (see Table 3 (Tab. 3) in the section on clinical manifestations). 

**Recommendations**

- A serology test should be conducted if MEM is suspected.
- If the serology test is negative and clinical suspicion of MEM persists, further clarification is needed by means of direct molecular detection or direct detection by culture from biopsy material. (Strong consensus: 13/0/0)

#### 7.3.4 Borrelial lymphocytoma 

Diagnoses must be confirmed by serological antibody detection (Table 4 (Tab. 4)), whereby in most cases antibodies against *B. burgdorferi* can be detected [47], [76], [139]. When findings remain inconclusive, patients shall be referred to a dermatologist so that the pathogen can be detected by PCR or, if necessary, by culture. Two skin biopsies should be taken from the altered patch of skin (see 3.2.2) – one in a 4% formalin solution for the histological assessment, and one in a sterile, physiological saline solution for the culture and PCR test. 

*B. burgdorferi* can be detected by PCR and culture. Although there is limited data on the sensitivity of the methods with respect to borrelia lymphocytoma, PCR detection has a success rate of 70% [81], [139]. 

**Recommendations**

- If there is an unambiguous clinical presentation of borrelial lymphocytoma and a positive serology, further microbiological tests are not required. (Strong consensus: 13/0/0)
- If there is an unambiguous clinical presentation of borrelial lymphocytoma, antibiotic treatment is to begin immediately. (Strong consensus: 13/0/0)
- If the clinical presentation is not unambiguous and the serology is negative, further tests (primarily histology, molecular biology, possibly culture) are to be conducted to determine the differential diagnosis. (Strong consensus: 13/0/0)

#### 7.3.5 Acrodermatitis chronica atrophicans 

If there is a clinical suspicion of ACA, a Borrelia serology test should be carried out first (Table 4 (Tab. 4)). High antibody levels in the IgG screening test, combined with a broad spectrum of borrelial-specific bands in the IgG blot or similar tests (see section 4.1.1), are indications of ACA. A negative IgG serology rules out, with high certainty, ACA in immunocompetent patients. 

In a large cohort of 693 Slovenian patients diagnosed with ACA within a period of 28 years, all patients were found to have highly elevated levels of IgG antibodies, and 30% also had elevated levels of IgM antibodies [3]. The detection of plasma cells in a skin biopsy is an additional indicator that helps to confirm the diagnosis. 

In ambiguous cases the patient should be referred to a dermatologist for differential diagnosis. If uncertainty remains, two skin biopsies should be taken from the altered patch of skin (see 3.2.2) – one in a 4% formalin solution for the histological assessment, and one in a physiological saline solution for the culture and/or PCR test. In the case of ACA, *B. burgdorferi* DNA can be detected in around 70% of patients.

**Recommendations**

- If there is clinical suspicion of ACA, the diagnosis shall be confirmed by a serology test. (Strong consensus: 13/0/0)
- High IgG antibody levels in the screening test in combination with a broad band pattern in the IgG immunoblot support the suspected clinical diagnosis. (Strong consensus: 13/0/0)
- A negative Borrelia serology rules out ACA with a high degree of certainty in immunocompetent patients. (Consensus: 12/0/1)
- Histological confirmation of the diagnosis should be carried out. (Strong consensus: 13/0/0)
- When the clinical presentation is ambiguous, further diagnostic clarification should be carried out with a biopsy and subsequent histological assessment. If the findings are unclear, direct detection by culture and molecular methods is recommended. (Strong consensus: 13/0/0)

#### 7.3.6 Ambiguous dermatological pathologies for a suspected case of Lyme borreliosis

**Recommendations**

- If a cutaneous manifestation of Lyme borreliosis is suspected and the clinical presentation is ambiguous, a skin biopsy with a fine-tissue (histology) assessment should be carried out together with direct pathogen detection by culture and molecular methods. (Strong consensus: 13/0/0)
- If the clinical presentation is ambiguous and the serological and histological assessments are negative, an enhanced interdisciplinary diagnosis should be performed to clarify the differential diagnosis. (Strong consensus: 13/0/0)

### 7.4 Non-recommended diagnostic approaches 

In addition to the traditional diagnostic methods listed above, which are used for a suspected case of Lyme borreliosis, the literature describes a range of other diagnostic methods, some of which have not been fully evaluated. This includes the immunohistochemical detection of *B. burgdorferi* in biopsies, antigens in blood and urine, a CD57-positive/CD3-negative lymphocyte subpopulation, dark-field microscopy, ‘xenodiagnosis’ (hard-bodied tick larvae feed on the blood of patients with suspected Lyme borreliosis; the larvae are then analysed for Borrelia) as well as functional tests to test cellular immunity (lymphocyte transformation tests (LTT), cytokine detection). Currently there is a lack of scientific studies that prove there is a diagnostic benefit. In particular, LTTs should not be used as the tests that are currently available have a poor specificity. A current systematic review and an independent study on cellular testing show that no reliable evidence can be derived from the scientific literature to justify the use of these tests in routine diagnostic testing [140], [141].

**The following approaches should not be used to diagnose cutaneous manifestations of Lyme borreliosis:**

- The immunohistochemical detection of Borrelia in tissue (strong consensus: 13/0/0)
- PCR in serum and urine (strong consensus: 13/0/0)
- Lymphocyte transformation tests (LTT), enzyme-linked immunospot assay (ELISPOT) and/or the detection of specific cytokines (consensus: 12/1/0)
- The detection of Borrelia in feeding ticks (consensus: 12/0/1)
- The detection of Borrelia antigens in patient samples (strong consensus: 13/0/0)
- Direct microscopy to detect Borrelia in patient samples (strong consensus: 13/0/0)
- The detection of circulating immune complexes (strong consensus: 13/0/0)
- The detection of so-called L-forms or spheroblasts (strong consensus: 13/0/0)
- Rapid antibody tests (point-of-care tests (POCT)) due to lack of sensitivity (18–32%) (strong consensus: 13/0/0)
- “Visual Contrast Sensitivity Test” (VCS test or grey shade test) (strong consensus: 13/0/0

### 7.5 Quality control and quality assurance 

According to the current guideline of the German Medical Association, diagnostic laboratories must participate twice a year in external quality assessment (EQA) schemes for serological infectious disease testing. This applies to serological antibody detection and to direct molecular detection of Borrelia when Lyme borreliosis is suspected. INSTAND e.V. has been carrying out such EQA schemes for many years. The results of these EQA schemes reveal an extensive heterogeneity in the test systems currently on the market. The pass rates for conventional serological and molecular test systems taken from meta-analyses indicate that, even though the immunoassays and molecular tests have good analytical pass rates, a clinical diagnostic interpretation of result constellations often proves difficult and can complicate medical care in everyday clinical practice [16], [103]. Thus, when Lyme borreliosis is suspected, diagnostic infectious disease testing is to be conducted in laboratories that meet the diagnostic standards as per the guidelines of the medical societies and the guideline of the German Medical Association. These laboratories must successfully participate in external quality assessment schemes (EQAs) on a regular basis. Physicians treating patients with Lyme borreliosis should enquire about and ensure that these conditions are met by the laboratories charged with carrying out their diagnostic testing. The laboratories specialising in Lyme borreliosis testing and the National Reference Centre for Borrelia at the Bavarian Health and Food Safety Authority in Oberschleissheim should be consulted in the event that there are questionable result constellations or implausible test results. 

**Recommendation**

- Attending physicians shall be aware of whether their testing laboratory complies with the relevant diagnostic standards and has the necessary competencies. They shall also be aware of the extent to which the diagnostic assays it uses conform to the guidelines. (Strong consensus: 13/0/0)

## 8 Treatment of cutaneous Lyme borreliosis

Recommendations for treating Lyme borreliosis have appeared in numerous European and American guidelines since 2004 (see Annex 2 Comparison of Guidelines and Therapies in Attachment 1).

Table 5 (Tab. 5) summarises the best evaluated antibiotic therapies listed in American and European guidelines. 

**Doxycycline and Amoxicillin** are the antibiotics of choice in all guidelines. 

Both antibiotics are very effective in the dosages listed in Table 5 (Tab. 5) and are usually tolerated well [1], [142]. Gastrointestinal complaints can occur during doxycycline treatment. It is particularly important that doxycycline not be taken together with dairy products, dairy substitutes or drinks rich in Ca2+. Furthermore, patients should be informed of the risk of phototoxic skin reactions, particularly in the summer, and use sunscreen when taking the antibiotics.

During treatment with amoxicillin, non-allergenic skin exanthemas can frequently appear on the torso on day 8. 

Treatment can continue if the exanthema is mild. If itching occurs, symptoms can be treated with antihistamines and skin care products. Corticosteroids are not necessary. 

Of the oral cephalosporins, only **cefuroxime axetil** has demonstrated an efficacy that is comparable to that of doxycycline and amoxicillin [143]. The absolute bioavailability of cefuroxime axetil is comparatively low (40–45%). The best absorption is achieved when taken directly after a meal.

Other 1^st^ and 2^nd^ generation cephalosporins are not adequately effective [144]. 

In the case of an early disseminated infection, intravenous treatment with ceftriaxone does not achieve any better results than treatment with oral doxycycline [145].

Of the macrolides, **azithromycin** has proven to be sufficiently effective [1], [101], [142], [146], [147], [148]. The long tissue half-life period is advantageous because of the long generation time of Borrelia. The efficacy of **clarithromycin** is deemed controversial. Clarithromycin was compared to amoxicillin in a recent randomised open comparative study of children with erythema migrans and was found to be equally effective [1], [142], [149]. Roxithromycin is not adequately effective. Erythromycin is no longer the treatment of choice due to its unreliable absorption and indications of resistance [150]. 

Treatment with oral penicillin V is controversial. Austrian, Swedish and Slovenian studies show that it is adequately effective [101], [151], [152], [153]. Cochrane’s network meta-analysis has shown that oral penicillin V is just as effective as amoxicillin and doxycycline [1]. The dosage for adults is 3x1 million IU/day and for children 100,000 IU/kg/day for 14 days [101].

Minocycline is effective and authorised for the treatment of Lyme borreliosis. However, it cannot be recommended due to the increased risk of side-effects relating to the central nervous system and liver toxicity. Cochrane’s network meta-analysis has also shown that there is inadequate data, as there is only one comparative study of minocycline and penicillin V from 1996 [154].

**It is particularly important that dose and treatment duration are adhered to.**

**Early cutaneous manifestations** should be treated for 10–21 days (Table 5 (Tab. 5)). The length of treatment depends on the duration and severity of the clinical symptoms; 10–14 days of treatment is usually sufficient for solitary erythema migrans without general symptoms. In a comparative study, Stupica et al. [155] evaluated the outcome of treating localised erythema migrans with doxycycline for 10 versus 14 days. No difference was found in the treatment outcomes of the erythema. In both treatment groups, symptoms did not persist longer or more frequently after the end of treatment than in healthy subjects. Treatment should last 21 days if there are indications of Borrelia dissemination (such as flu-like symptoms), or in the case of multiple erythemata migrantia and borrelial lymphocytoma.

Oral doxycycline or amoxicillin treatment for 30 days is usually sufficient for **late cutaneous manifestations** (acrodermatitis chronica in the oedematous-infiltrative or atrophic stages) without neurological involvement [3], [91], [154]. If neurological symptoms are also present, intravenous treatment with penicillin G or the 3^rd^ generation cephalosporins ceftriaxone or cefotaxime may be necessary (see the AWMF S3 guideline on neuroborreliosis [2]). To date there are no major controlled treatment studies on ACA.

The cure rate – defined as a restoration of the body to its original physical condition with regression of the disease-specific symptoms after successful treatment – is between 95% and 100% when the early localised and disseminated manifestations are treated in time [156], [157].

Treatment failures with pathogen detection after treatment are rare if treatment is carried out *lege artis* [54], [158]; isolated cases have been reported [121], [123], [159], [160], [161], [162]. Two major studies have shown that, in all cases, a new infection with another Borrelia strain was the cause of a renewed Lyme borreliosis infection [163], [164]. In a small case series, treatment failure was observed when there was immunosuppression with a TNF-α inhibitor [165].

In individual cases of late cutaneous manifestations where there is continued clinical and biopsy evidence of a persisting infection, treatment should be repeated with a different class of substance.

To date, there is no evidence of the development of secondary antibiotic resistance of *B. burgdorferi* to the antibiotics recommended in the guidelines [166], [167], [168], [169].

If the disease remains untreated for a longer period of time, there is an increased risk of persistent symptoms and defective healing of the skin, joints and nervous system increases [3].

It is a matter of controversy whether repeated antibiotic treatment makes sense for patients with persisting symptoms. According to published randomised controlled trials (RCT), long-term antibiotic treatment is less than promising [170], [171], [172], [173], [174], [175].

A European RCT that was published in 2016 (PLEASE Study) looked at 280 patients whose symptoms persisted for more than 2 years after their Lyme borreliosis was treated with antibiotics (78 patients after erythema migrans, 15 patients after meningoradiculitis) and 153 seropositive patients with borreliosis-related symptoms after a tick bite. The study compared the health-related effects of a 2-week versus 14-week round of antibiotics. Initially, all patients who had previously received antibiotic treatment received 2 g of ceftriaxone i.v. for 2 weeks. The patients were then randomly assigned to 3 groups. Group 1 received 200 mg/d of doxycycline p.o. for 12 weeks, group 2 received 2x500 mg of clarithromycin plus 2x200 mg/d of hydroxychloroquine for 12 weeks, and group 3 received a placebo for 12 weeks. Treatment success was based on a health-related quality of life after 14 weeks and then up to 52 weeks using the RAND 36 Health Status Inventory. The sum score improved equally after treatment in all 3 groups without significant differences; the assessment of the quality of life remained below that of the general population in all 3 groups. No difference in treatment success could be found between the short-term treatment and the two long-term treatments. Patients receiving the long-term treatment had considerably more antibiotic-related side-effects (primarily photosensitivity (18.6%) and nausea (10.5%) in connection with doxycycline, and primarily nausea (10.4%), diarrhoea (9.4%) and allergic exanthemas (8.3%) in connection with clarithromycin/hydroxychloroquine). Vision problems were most frequently reported in the placebo group (10% of patients) [176], [177].

### 8.1 Treatment during pregnancy and nursing 

Oral treatment with amoxicillin p.o. is recommended during pregnancy and nursing. Alternatively, penicillin G and ceftriaxone can be administered i.v. [178], [179]. If the patient has a proven penicillin allergy, azithromycin or cefuroxime axetil can be prescribed after strong indication. Ceftriaxone can be administered intravenously under clinical supervision since, with 3^rd^ generation cephalosporins, the risk of a cross allergy with penicillin is only around 1% [180].

### 8.2 Treating children 

Children can be treated with **doxycycline** at a dose of 4 mg/kg BW/day up to a maximum dose of 200 mg/d from the age of 9 (>8 years) once enamel formation is complete. Even today, prescribing doxycycline to treat erythema migrans in children under 9 years of age is not recommended [181]. For children under 8, the treatment of choice is 50 mg/kg BW/d of **amoxicillin** (Table 5 (Tab. 5)). Taking it the required 3 times a day can be difficult for kindergarten- and school-aged children. 

Alternatively, 30–50 mg/kg BW/d of **cefuroxime axetil**, 5–10 mg/kg BW/d of **azithromycin**, or 15 mg/kg BW/d of **clarithromycin** in two daily doses can be prescribed [149].

### 8.3 Treatment adherence

In order to improve **treatment adherence/therapy compliance**, the patient should be informed before beginning the treatment of the aspects of taking prescribed antibiotics and the potential risk of undesired effects. 

A frequent cause of treatment failure is when doxycycline is taken incorrectly. It should be noted that absorption can be impaired by 2- or 3-valent cations such as aluminium, calcium (milk, dairy products and fruit juices containing calcium, water with a high mineral content), magnesium in antacids, iron preparations, as well as activated charcoal and cholestyramine. Therefore, there should be a 2-to-3-hour interval between taking the antibiotic and ingesting the medicine or food. 

Another cause of treatment failure is when the antibiotic is taken irregularly e.g. skipping the midday dose in the case of amoxicillin, or not long enough e.g. due to a worsening of symptoms caused by a Herxheimer reaction, gastrointestinal complaints, or phototoxic skin reactions due to increased sensitivity to light from doxycycline. In addition, treatment failure was reported in a case series where there was immunosuppression with a TNF-α inhibitor [165].

In the case of a disseminated infection, patients should be informed about the possible occurrence of a **Herxheimer reaction** with a flare up of the erythema, which occurs in approx. 10% of cases. Approx. 2% of patients experience a feeling of unwellness and a rise in temperature within 24 hours of taking the antibiotics [88], [149]. Occasionally this reaction is delayed. This is a temporary immunological reaction caused by the upregulation of proinflammatory cytokines and can be treated, for example, with non-steroidal anti-inflammatory drugs (NSAID). Cortisone treatment is not necessary. The patient should continue to take the antibiotic. 

**Key statements and recommendations for the treatment of cutaneous Lyme borreliosis**

**Lyme borreliosis should be treated with antibiotics.**

- Doxycycline or amoxicillin p.o. are the treatments of choice for **cutaneous manifestations**. (Consensus 13/0/1)
- Treatment alternatives **for cutaneous manifestations** are cefuroxime, azithromycin, possibly also clarithromycin p.o. (Strong consensus 14/0/0)
- See the recommendations of the other medical associations for the antibiotic treatment of patients with cutaneous Lyme borreliosis with neurological or cardiological manifestations. Ceftriaxone i.v., cefotaxime i.v., penicillin G i.v. or doxycycline p.o. may be considered. (Strong consensus 14/0/0)
- The treatment of early manifestations of cutaneous Lyme borreliosis should last 14–21 days (with the exception of azithromycin 5–10 days; doxycycline 10–14 days in the case of solitary erythema migrans). (Strong consensus 14/0/0)
- The treatment of late cutaneous manifestations should last 30 days. (Strong consensus 14/0/0)
- Antibiotic treatment should not generally extend beyond the recommended time period. (Consensus: 13/0/1)
- Treatment can be extended in individual cases depending on the clinical course of the disease and after re-evaluating the diagnosis. (Strong consensus: 14/0/0)
- Treatment should be repeated with a different class of substance in individual cases of late cutaneous manifestations if there is clinical or biopsy evidence of a persisting infection. (Consensus: 13/1/0)
- The diagnosis should be re-evaluated if the cutaneous symptoms persist or progress despite guideline-compliant antibiotic treatment. (Strong consensus: 14/0/0)

**Recommendations for treating cutaneous Lyme borreliosis during pregnancy**

- Amoxicillin p.o. is the treatment of choice during pregnancy. (Strong consensus: 14/0/0)
- Treatment alternatives during pregnancy or while nursing are penicillin G i.v. or ceftriaxone i.v. (Strong consensus: 14/0/0)
- Cefuroxime p.o., ceftriaxone i.v., cefotaxime i.v. or azithromycin p.o. should be prescribed if the patient is allergic to penicillin. (Strong consensus: 14/0/0)

**Treatment recommendation for cutaneous Lyme borreliosis in children**

- Amoxicillin p.o. is the treatment of choice for children under 8 years of age. (Strong consensus: 14/0/0)
- Children 9 years and older can receive doxycycline p.o. (Strong consensus: 14/0/0)
- Azithromycin, clarithromycin or cefuroxime p.o. are treatment alternatives for children. (Strong consensus: 14/0/0)

See the guideline report for the **dissenting opinion** of the Borreliosis and FSME Association of Germany on treatment [25].

### 8.4 Persisting symptoms after treatment/post-treatment Lyme disease syndrome (PTLDS)

Inflammatory reactions can persist and symptoms such as fatigue, joint and muscle pain, headaches, a general feeling of unwellness, irritability or paraesthesia can last for months, even following guideline-compliant antibiotic treatment. If the non-specific symptoms persist for more than 6 months, this is referred to by some authors as Post Lyme Syndrome (PLS) or Posttreatment Lyme Disease Syndrome (PTLDS) [173], [182]. So-called PTLDS is a syndrome that has yet to be generally defined scientifically and is, therefore, not yet universally accepted. It can be differentiated diagnostically from confirmed late manifestations of Lyme disease, symptoms resulting from the persistence of reproducible pathogens, and dysfunctional healing. Repeated and long-term antibiotic treatment does not provide any proven benefit [183], but is associated with the risk of considerable side effects, some of which can be life-threatening [170], [171], [172], [174]. The issue of PTLDS is discussed in detail in the S3 guideline on neuroborreliosis [2] (online: https://www.dgn.org/leitlinien).

A controlled study of patients with erythema migrans, in which an age- and sex-matched control group was simultaneously studied, found that there was no increased incidence of post-therapeutic symptoms compared to the control group [156]. 

It is crucial that the complaints of patients with new or persisting symptoms after guideline-compliant antibiotic treatment are taken seriously, further diagnostic testing is performed on a case-by-case basis, and that any further treatment is carried out after careful consideration of the risks and benefits [183]. The need for research into PTLDS remains exceedingly high. 

### 8.5 Procedure when skin changes and symptoms persist after antibiotic treatment 

A false primary diagnosis is a common reason why there are persisting skin changes and symptoms following antibiotic treatment 2009 [184].

In the case of a clinically diagnosed erythema migrans and multiple erythema migrantia that do not heal within 6 weeks, a differential diagnosis of circumscribed scleroderma (morphea), granuloma annulare, sarcoidosis, erythema annulare et diutinum, tinea (with low epidermal involvement) or urticarial vasculitis should be considered. Patients should be referred to a dermatologist for further diagnostic testing. Borrelial lymphocytoma often heals very slowly over many months. According to investigations by Maraspin et al. on 85 patients, the average healing time was 28 days (7–270 days). The longer the borrelial lymphocytoma was present, the longer it took to heal [81]. If nodules persist for more than 1 year or if new nodules appear, a skin biopsy should be performed by a dermatologist for histological analysis and a Borrelia PCR. The differential diagnosis includes a cutaneous pseudolymphoma, a Jessner-Kanof lymphocytic infiltration, or a malignant lymphoma. 

It takes years following antibiotic treatment for skin changes to slowly regress in cases of acrodermatitis chronica atrophicans that have persisted for years. Atrophies of the skin, tissue and fat can be irreversible – especially in older people. This also applies to ACA-associated peripheral neuropathy. (See also the AWMF-S3 Guideline on Neuroborreliosis [2].) 

The differential diagnosis includes age-related skin atrophy, chronic thermal damage to the skin, e.g. chilblains and heat melanosis, as well as chronic venous insufficiency with stasis dermatitis.

Chronic neuropathic pain after adequate antibiotic treatment of acrodermatitis chronica atrophicans with peripheral neuropathy is treated in accordance with the DGN’s guideline on neuropathic pain (AWMF – guideline register no. 030/114 [185]). 

All patients with persisting symptoms that cannot be attributed to cutaneous Lyme borreliosis or can no longer be attributed to cutaneous Lyme borreliosis after antibiotic treatment, should undergo careful differential diagnosis by an appropriate specialist, above all for internal medicine (infectiology, rheumatology, cardiology, endocrinology) as well as for neurology, psychosomatics and psychotherapy, psychiatry and pain therapy, since chronic infectious diseases of a different aetiology, other internal diseases, autoimmune diseases, chronic pain syndromes, depressive and somatoform or somatic stress disorders may also be considered in the differential diagnosis and must be treated accordingly (AWMF S3 Leitlinie “Funktionelle Körperbeschwerden” [186]; [2], [183], [187]).

There are no clinical studies that specifically look at the (symptomatic) treatment of persisting symptoms following adequate antibiotic treatment of Lyme borreliosis.

**Recommendations for persisting symptoms following guideline-compliant treatment**

**If an erythema treated as erythema migrans or multiple erythema persists** (longer than 6 weeks), the patient shall be referred to a dermatologist for a differential diagnosis of circumscribed scleroderma (morphea), granuloma annulare, sarcoidosis, erythema annulare et diutinum, tinea or urticarial vasculitis. (Strong consensus: 12/0/0)

**If a lymphocytoma persists or progresses after treatment, **the patient shall be referred to a dermatologist for a differential diagnosis (cutaneous pseudolymphoma, Jessner’s lymphocytic infiltration or malignant lymphoma). (Strong consensus: 12/0/0)

**If an acrodermatitis chronica atrophicans persists after treatment,** the patient shall be referred to a dermatologist for further consultation and for a differential diagnosis (age-related skin atrophy, chronic thermal damage to the skin e.g. chilblains and heat melanosis, chronic venous insufficiency with stasis dermatitis). (Strong consensus: 12/0/0)

## 9 Prophylaxis

### 9.1 Preventing tick bites 

The best prophylaxis is to prevent tick bites by wearing clothing that covers the body and to carefully check the skin, including the scalp under the hair, after spending time outdoors. This is particularly important for children, who are at increased risk when playing outdoors between spring and autumn. 

Insect repellents that are effective against ticks, e.g. diethyl-meta-toluamide (DEET), icaridin (1-(1-methylpropyl carbonyl)-2-(2-hydroxyethyl)piperidine) and ethyl butylacetylaminopropionate (EBAAP, IR 3535) can also be used, but only have a limited effectiveness of up to 4 hours [188], [189], [190]. 

Clothing that is impregnated with permethrin is also available, although no studies are available on its ability to repel *I. ricinus* or its long-term effects on humans. 

### 9.2 Preventing Lyme borreliosis 

It is very important to** remove ticks early**, before they have become engorged with blood. The risk of Borrelia transmission increases with the length of the time the tick feeds [24]. The time the *I. ricinus* needs to feed before there is a risk of transmission to humans has not yet been sufficiently investigated. It is important to emphasise that results from the USA, which assume transmission after at least 24 hours of feeding – but with a different vector and only *B. burgdorferi* s.s. – are not transferable to Europe. Therefore, every tick bite must be regarded in principle as potentially infectious, even if the probability of transmission increases with the length of time of the feeding act. 

After spending time in a garden, park, field, forest or meadow where contact with ticks is possible, the body should be checked the same evening for ticks. Showering alone is not enough. The tick often remains on the body while it searches for a suitable location, e.g. back of the knees, armpits, between the toes, groin, genitals, in children often under the hair on the scalp, or neck. 

Ticks should be removed immediately using tick tweezers or tick cards to prevent the transmission of Borrelia. If parts of the feeding apparatus remain in the skin, they can be removed later using a needle or curettage [182]. If the head or the feeding apparatus remains in the skin, there is no harm with regard to the transmission of the Borrelia. In the case of fully engorged nymphs and adult ticks, the body of the tick should not be squeezed in order to prevent the possible transmission of the Borrelia. 

Examining a tick that has been removed from the skin for Borrelia is not useful, as the detection of Borrelia in the tick is not sufficiently predictive of the transmission of the Borrelia to the host and the development of disease. 

After the removal of a tick, patients should be informed about the **need for observing**, in particular, the bite site over the next 6 weeks (Annex 1: Patient information after a tick bite in Attachment 1). 

### 9.3 Prophylactic treatment after a tick bite 

According to an American study, the risk of infection can be reduced by taking a one-time prophylactic dose of 200 mg doxycycline after a tick bite (effectiveness of 87%) [191], [192]. However, the findings should be interpreted with caution, as only one follow-up took place after 6 weeks. Thus, no statement can be made about whether this is sufficiently effective with respect to late infections. 

A recent European study – an open-label RCT with participation via the internet – looked at 1,689 subjects. It analysed participants >8 years of age, doxycycline 1x200 mg within 72 h after tick removal, no tick bite before or in the following 3 months, completed questionnaire after 1 week, 1 month and then every 3 months up to 18 months and no evidence of Lyme borreliosis when entering the study. A total of 29 cases (28 erythema migrans and one acrodermatitis chronica atrophicans) were found, 10 out of 1,041 (0.96%) in the prophylaxis group and 19 out of 648 (2.9%) in the group without prophylaxis. This results in a number needed to treat (NNT) of 51 (95%-CI 29–180) to prevent one case of Lyme borreliosis, corresponding to a relative risk reduction of 67% (95%-CI 31–84%) [193]. The NNT could be reduced to around 10 when other parameters were included, such as the tick’s feeding conditions or a Borrelia PCR from the tick, but such procedures – including tick removal, dispatch to a laboratory with the appropriate expertise, phenotypic and molecular tests – in order to receive a prophylaxis within 72 h is not feasible. The authors conclude that prophylaxis in Europe is as effective as in the USA, that internet studies allow larger numbers of participants and that the inclusion of tick properties in the decision-making process could further increase efficacy. 

In light of the low risk of infection, doxycycline would have to be unnecessarily administered many times in order to prevent a potential infection. According to projections for infection risk in endemic areas, 40–125 prophylaxes would have to be administered to prevent 1 infection [194]. Impact on the intestinal flora and possible development of resistance through the frequent administration of prophylaxes is conceivable. For this reason, oral doxycycline prophylaxis is not recommended in Europe. 

The prophylactic use of antibiotic creams is also controversial. Animal studies with azithromycin cream reveal good prophylactic efficacy [195], [196]. A placebo-controlled study on efficacy in humans did not find sufficient efficacy, which is why this prophylactic measure cannot be recommended at present [197].

**Recommendations for infection prophylaxis**

**Clothing that covers the body should be worn to prevent tick bites.**

- The use of tick repellents can be recommended to a limited degree.
- After spending time outdoors where contact with ticks is possible, the skin should be checked for ticks no later than that evening.
- Ticks should be removed early to prevent Lyme borreliosis.
- The site of the bite should be observed for up to six weeks.
  (Consensus: 12/0/1)

**Not recommended**

- The removed tick should not be analysed for Borrelia (Consensus: 12/0/1)
- Local or systemic prophylactic antibiotic treatment after a tick bite should not be carried out. (Consensus: 12/0/1)

### 9.4 Vaccines 

Currently there is no vaccine approved for use in humans.

A vaccine with lipidated recombinant Osp A was evaluated as part of large-scale studies in the USA and was found to have good efficacy [198], [199]. The vaccine was approved in the USA in 1999 but was withdrawn from the market by the manufacturer in 2002. The reasons for this are not of a medical nature. Reports of adverse vaccine reactions in genetically predisposed individuals have been refuted by several qualified studies [200], [201], [202]. This monovalent vaccine is not suitable for Europe as it only protects against infection with *B. burgdorferi* sensu stricto and not against the genospecies *B. afzelii* and *B. garinii*, which are frequently found in Europe. A polyvalent OspA vaccine is currently being developed for Europe [203], however approval is not expected in the foreseeable future. 

## Notes

### Guideline information

This is the English version of the German DGN S2k Guideline “Kutane Lyme Borreliose”, AWMF Register Number: 013/044 [204].

### Disclaimer 

The “guidelines” of the scientific medical societies are a systematically developed aid for physicians to reach decisions in specific situations. They are based on current scientific knowledge and good practice and ensure greater safety in medicine. They also take economic factors into consideration. The “guidelines” are not legally binding for physicians and therefore have neither a liability or liability releasing function. 

The AWMF prepares and publishes the guidelines of the medical societies with great care – however the AWMF assumes no **liability** for accuracy – **particularly with regard to dosage information**.

### Procedure for forming a consensus

The guideline was drawn up using a modified Delphi process and voted on in an extended consensus conference of the Interdisciplinary S3 Guideline Group, moderated by Prof Ina Kopp, Head of the AWMF Institute for Medical Knowledge Management. 

It was adopted by the 22 participating medical societies and patient organisations.

### Competing interests

See Attachment 2.

## Supplementary Material

Annexes

Competing interests

## Figures and Tables

**Table 1:**
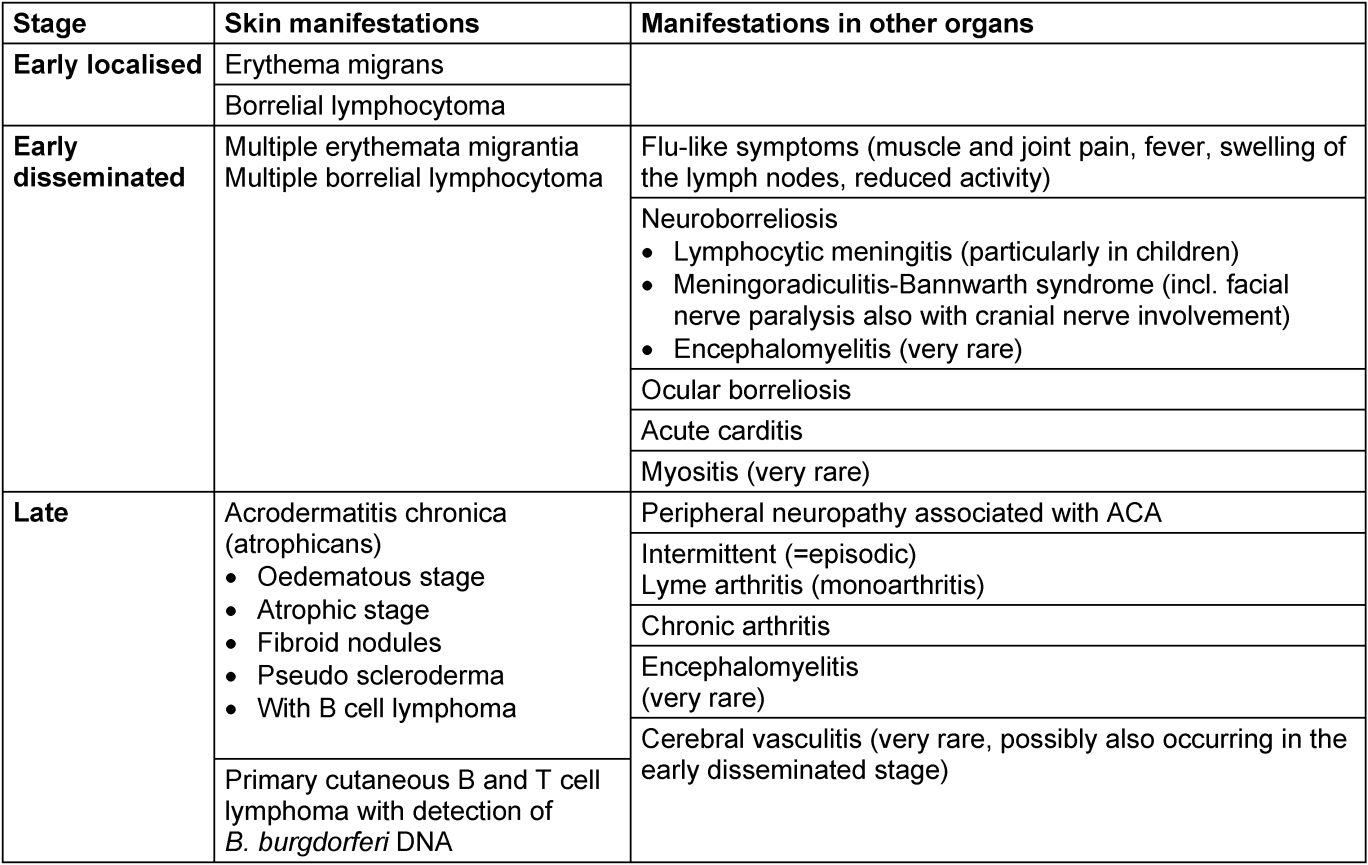
Clinical manifestations of Lyme borreliosis

**Table 2:**
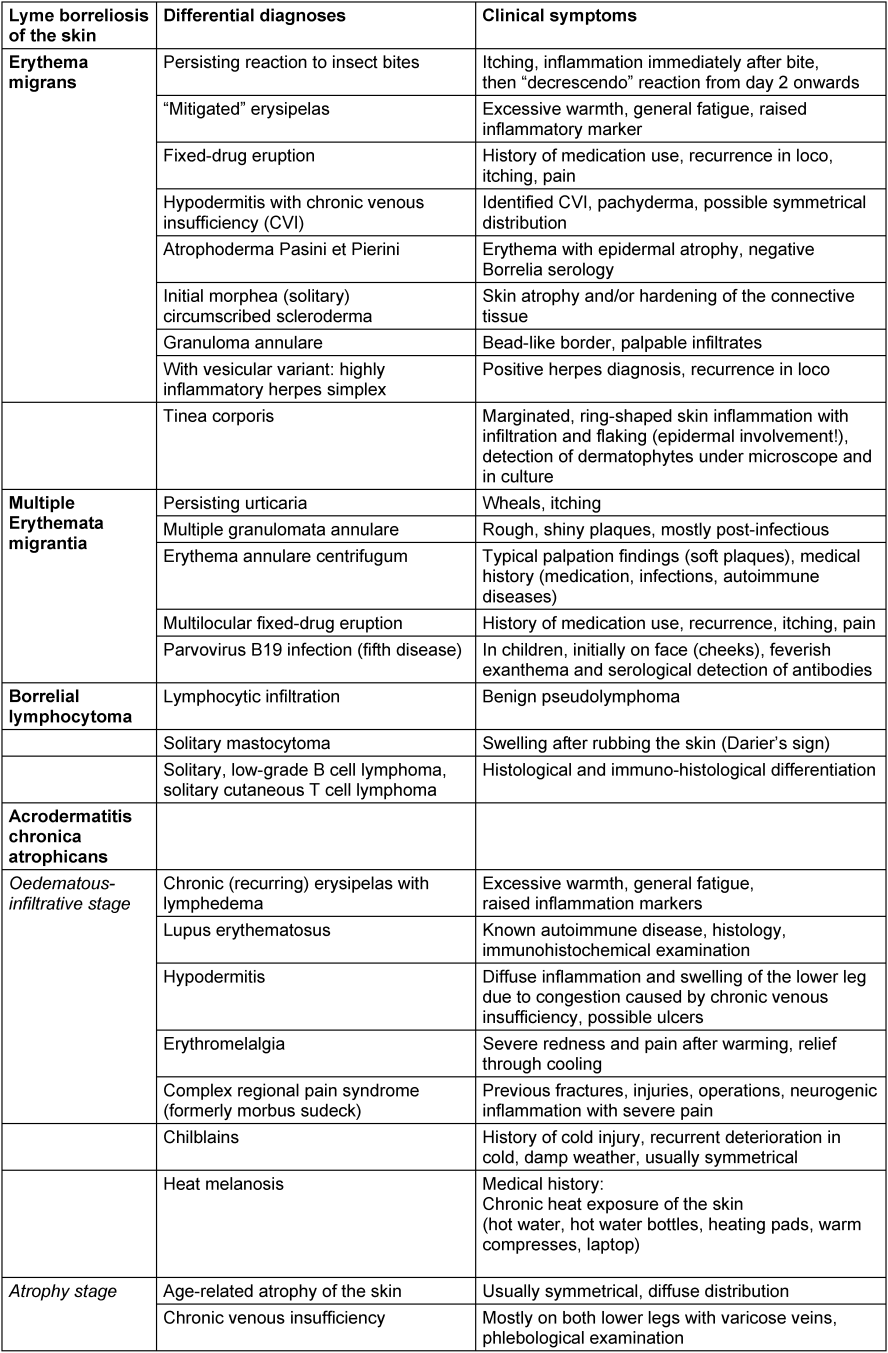
Clinical differential diagnoses of cutaneous Lyme borreliosis

**Table 3:**
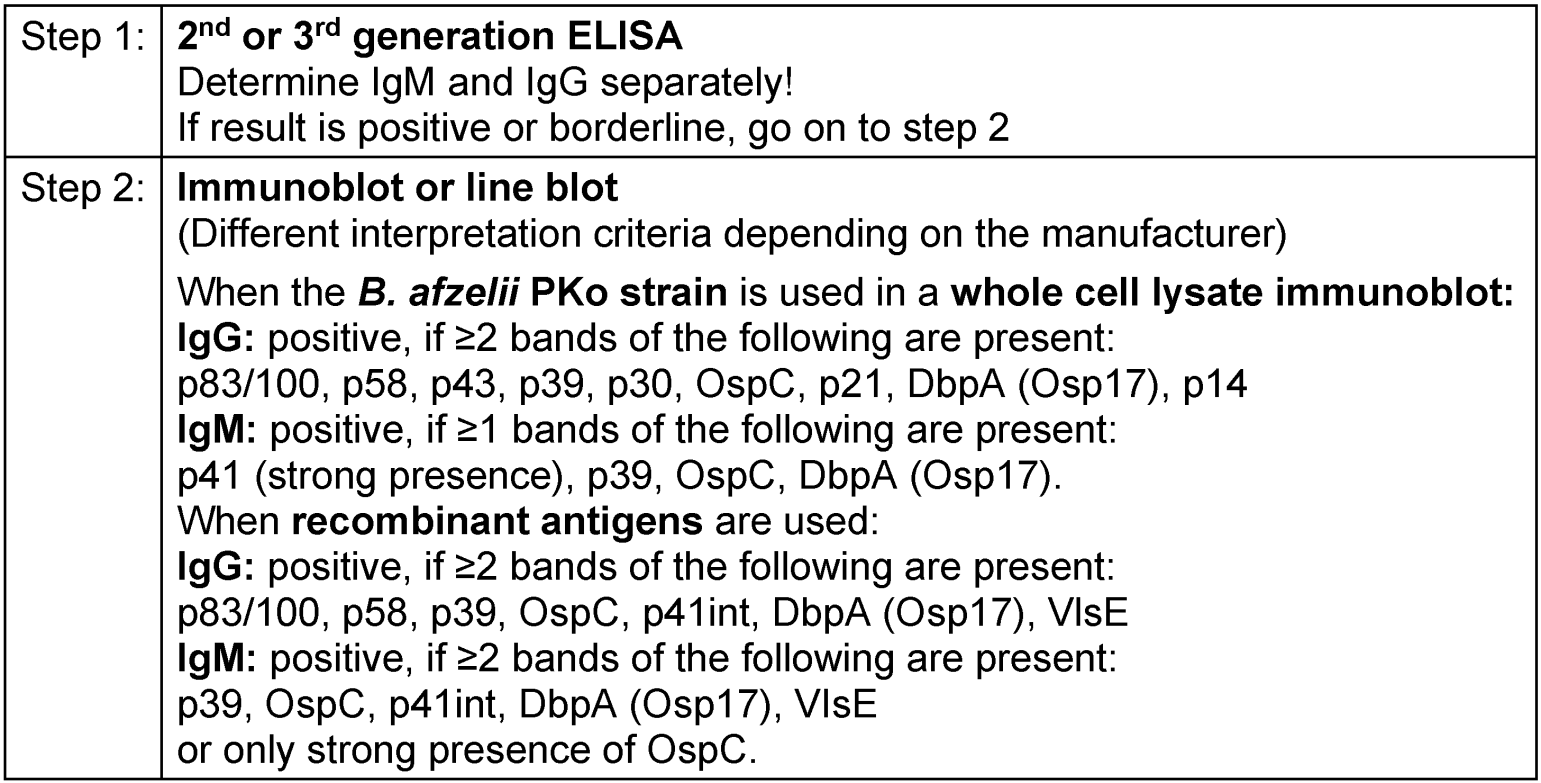
Step-by-step approach to serology testing (as per MIQ 12 and DIN 58969-44 2005-07)

**Table 4:**
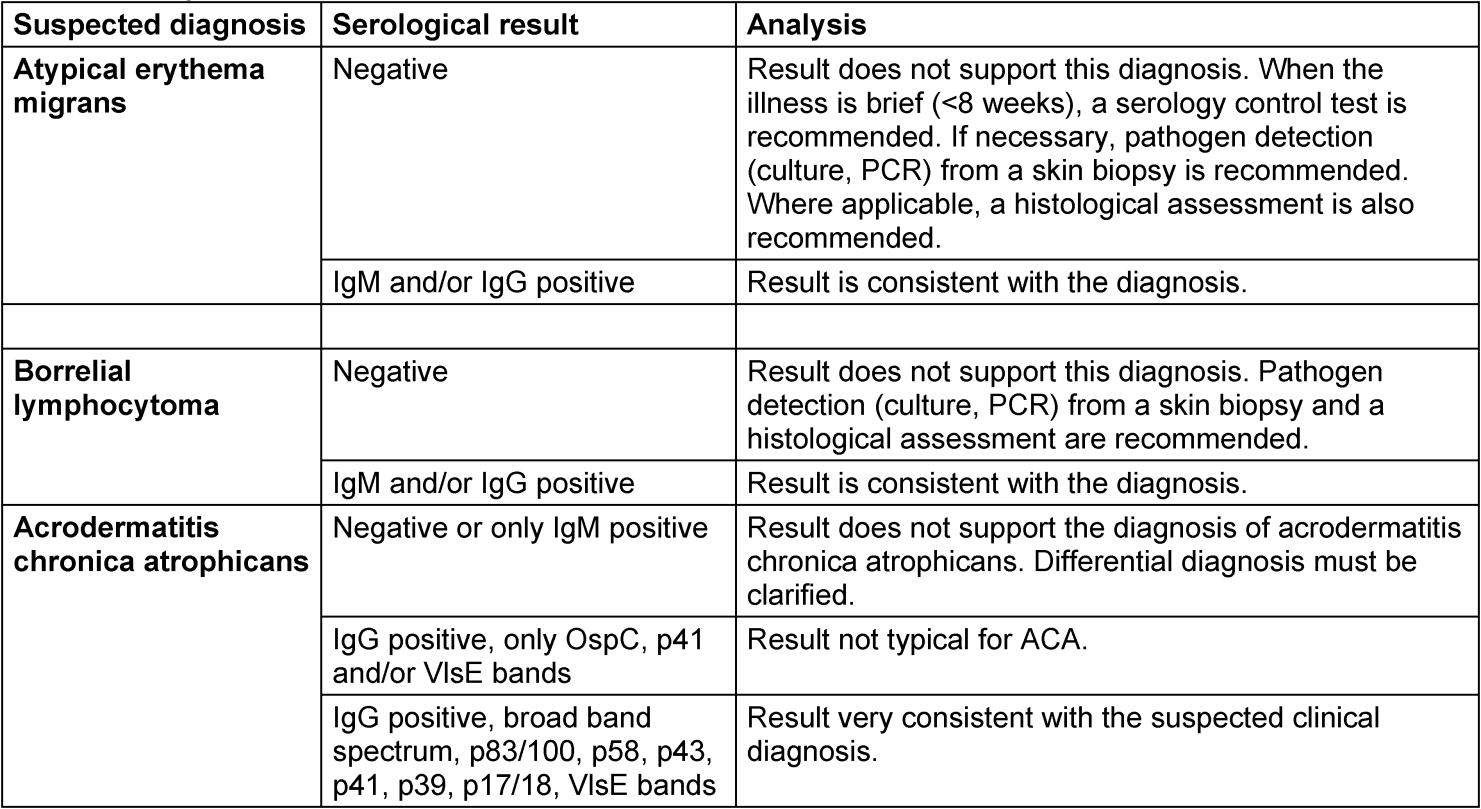
Interpretation of the constellations of the serological results

**Table 5:**
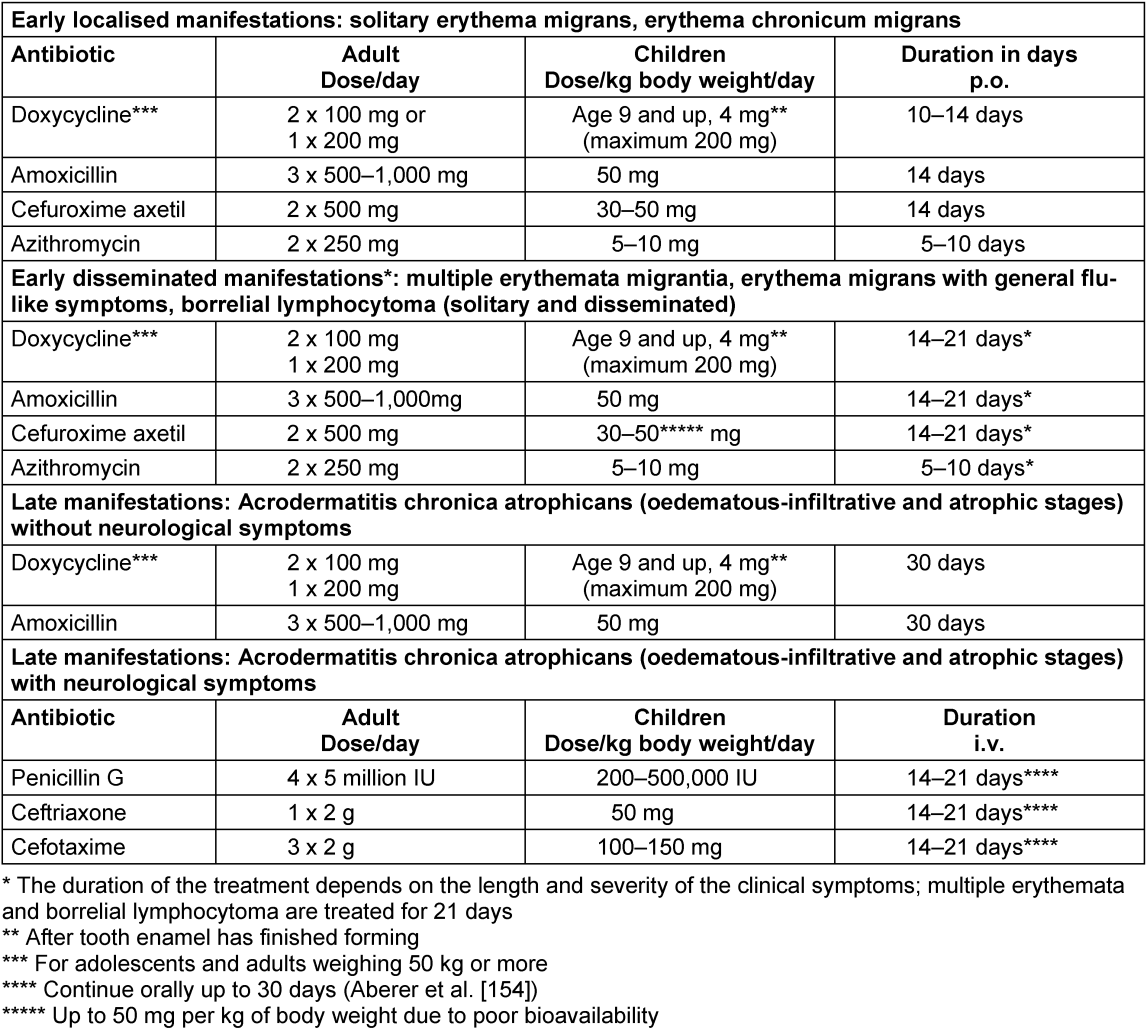
Treatment recommendations for cutaneous Lyme borreliosis
