# Supplementary material for: Cutaneous Lyme borreliosis: Guideline of the German Dermatology Society
Source: Ger Med Sci. 2025 Oct 9;23:Doc12. doi: 10.3205/000348 (PMC12584192; doi:10.3205/000348)
Supplement: Annexes [file GMS-23-12-s-001.pdf]

Attachment 1: Annexes

Annex 1: Patient information on what to do in the event of a tick bite

1. **Remove the tick as quickly as possible.**
   Ticks can be removed by the person who has been bitten. Special tick tweezers or tick cards are best suited, however in situations where these instruments are not available (e.g. camping, hiking trips), it is more important to prioritise the early removal of ticks without a special instrument.
   Depending on the instrument, slowly and patiently pull or push the tick out of the skin without twisting it or pre-treating it with oil or glue. Avoid squeezing the tick's body.
   If part of the feeding apparatus remains in the skin (often misinterpreted as the "head"), it can be removed with a sterile needle or curette, or it can be removed by a physician. If the feeding apparatus remains in the skin, there is no danger that the Borrelia will be transferred.
2. **Carefully examine your body and especially the heads of children for more ticks.**
3. **Observe the skin around the site of the bite for 6 weeks.** Any redness caused by the tick saliva, which appears immediately after the bite, will disappear within several days. If reddening **reappears** or if the initial reddening increases to ≥5 cm, a doctor should be consulted. This may be **erythema migrans (migrating rash)**, which is an early manifestation of Lyme borreliosis.
4. **If there is a typical migratory rash in the area around the tick bite, antibiotic treatment should be initiated, preferably with doxycycline (in children aged 9 and up) or with amoxicillin**, even if no blood test has been carried out or if no antibodies are detectable yet in the blood.
5. The dissemination of the Borrelia through the blood – even without a reddening of the skin – is recognisable by a flu-like feeling without respiratory symptoms. They may be the precursor of organ disease, e.g. of the joints or nervous system. In this case, consult a physician who will decide whether a blood test for Borrelia antibodies is necessary.
6. Lyme borreliosis can be completely cured in the early stages with guideline-compliant antibiotic treatment, thus preventing late manifestations.
7. It is not advisable to test the tick for Borrelia, since a positive result does not mean that the Borrelia will have been transmitted to the skin or that they will cause an infection if they have been transmitted. A negative result does not rule out transmission.
8. Only a small proportion of people infected with Borrelia become ill! This is why prophylactic oral antibiotic treatment is not recommended.

Attachment 1 to: *Hofmann H, Fingerle V, Rauer S, Hunfeld KP, Huppertz HI, Krause A, Consensus group. Cutaneous Lyme borreliosis: Guideline of the German Dermatology Society. GMS Ger Med Sci. 2025;23:Doc12.*
DOI: 10.3205/000348, URN: urn:nbn:de:0183-0003487

Annex 2: National and international guidelines on Lyme borreliosis

Germany

Deutsche Dermatologische Gesellschaft e.V. (DDG). S2k-Leitlinie Kutane Lyme Borreliose. Version 3.0. AWMF-Registernummer 013-044. Berlin: AWMF; 2023. Available from: https://register.awmf.org/de/leitlinien/detail/013-044

Deutsche Gesellschaft für Neurologie e.V. (DGN). S3-Leitlinie Neuroborreliose. Version 6.0. AWMF-Register-Nr. 030-071. Berlin: AWMF; 2024. Available from: https://register.awmf.org/de/leitlinien/detail/030-071

Deutsche Borreliose-Gesellschaft e.V. Diagnosis and Treatment of Lyme borreliosis. Revised 2nd edition. Deutsche Borreliose-Gesellschaft e.V.: Jena; 2011. Available from: https://www.borreliose-gesellschaft.de/?Wissenschaft#Wissenschaft/Leitlinien

Huppertz HI, Bartmann P, Heininger U, Fingerle V, Kinet M, Klein R, Korenke GC, Nentwich HJ; Committee for Infectious Diseases and Vaccinations of the German Academy for Pediatrics and Adolescent Health. Rational diagnostic strategies for Lyme borreliosis in children and adolescents: recommendations by the Committee for Infectious Diseases and Vaccinations of the German Academy for Pediatrics and Adolescent Health. Eur J Pediatr. 2012 Nov;171(11):1619-24. DOI: 10.1007/s00431-012-1779-4

Belgium

Belkhir L, Delaere B, De Loof G, De Munter P, Frippiat F, Jacobs F, Kabamba Mukadi B, Lacor P, Lernout T, Magerman K, Quoilin S, Rodriguez-Villalobos H, Rossi C, Saegeman V, Van S, Broucke D, Van der Linden D, Van Laethem Y, Vlieghe E, Vogelaers D. Borréliose de Lyme (infection à Borrelia). 2016. Available from: https://organesdeconcertation.sante.belgique.be/sites/default/files/documents/gids_lyme_borreliose_fr_march2017.pdf

Europe

Mygland A, Ljøstad U, Fingerle V, Rupprecht T, Schmutzhard E, Steiner I; European Federation of Neurological Societies. EFNS guidelines on the diagnosis and management of European Lyme neuroborreliosis. Eur J Neurol. 2010 Jan;17(1):8-16, e1-4. DOI: 10.1111/j.1468-1331.2009.02862.x

Brouqui P, Bacellar F, Baranton G, Birtles RJ, Bjoërsdorff A, Blanco JR, Caruso G, Cinco M, Fournier PE, Francavilla E, Jensenius M, Kazar J, Laferl H, Lakos A, Lotric Furlan S, Maurin M, Oteo JA, Parola P, Perez-Eid C, Peter O, Postic D, Raoult D, Tellez A, Tselentis Y, Wilske B; ESCMID Study Group on Coxiella, Anaplasma, Rickettsia and Bartonella; European Network for Surveillance of Tick-Borne Diseases. Guidelines for the diagnosis of tick-borne bacterial diseases in Europe. Clin Microbiol Infect. 2004 Dec;10(12):1108-32. DOI: 10.1111/j.1469-0691.2004.01019.x

Stanek G, Fingerle V, Hunfeld KP, Jaulhac B, Kaiser R, Krause A, Kristoferitsch W, O'Connell S, Ornstein K, Strle F, Gray J. Lyme borreliosis: clinical case definitions for diagnosis and management in Europe. Clin Microbiol Infect. 2011 Jan;17(1):69-79. DOI: 10.1111/j.1469-0691.2010.03175.x

Canada

Canadian Public Health Laboratory Network. The laboratory diagnosis of Lyme borreliosis: Guidelines from the Canadian Public Health Laboratory Network. Can J Infect Dis Med Microbiol. 2007 Mar;18(2):145-8. DOI: 10.1155/2007/495108

Switzerland

Nemeth J, Bernasconi E, Heininger U, Abbas M, Nadal D, Strahm C, Erb S, Zimmerli S, Furrer H, Delaloye J, Kuntzer T, Altpeter E, Sturzenegger M, Weber R; Swiss Society For Infectious Diseases; Swiss Society For Neurology. Update of the Swiss guidelines on post-treatment Lyme disease syndrome. Swiss Med Wkly. 2016 Dec 5;146:w14353. DOI: 10.4414/smw.2016.14353

Evison J, Aebi C, Francioli P, Péter O, Bassetti S, Gervaix A, Zimmerli S, Weber R. Borréliose de Lyme 1ère partie: épidémiotogie et diagnostic [Lyme disease Part I: epidemiology and diagnosis]. Rev Med Suisse. 2006 Apr 5;2(60):919-24.

Evison J, Aebi C, Francioli P, Péter O, Bassetti S, Gervaix A, Zimmerli S, Weber R. Borretiose de Lyme 2e partie: clinique et traitement [Lyme disease Part 2: clinic and treatment]. Rev Med Suisse. 2006 Apr 5;2(60):925-8, 930-4.

Evison J, Aebi C, Francioli P, Péter O, Bassetti S, Gervaix A, Zimmerli S, Weber R. Borréliose de Lyme 3e partie: prévention, grossesse, états d'immunodéficience, syndrome post-borréliose de Lyme [Lyme disease Part 3: prevention, pregnancy, immunodeficient state, post-Lyme disease syndrome]. Rev Med Suisse. 2006 Apr 5;2(60):935-6, 938-40.

France

Figoni J, Chirouze C, Hansmann Y, Lemogne C, Hentgen V, Saunier A, Bouiller K, Gehanno JF, Rabaud C, Perrot S, Caumes E, Eldin C, de Broucker T, Jaulhac B, Roblot F, Toubiana J, Sellal F, Vuillemet F, Sordet C, Fantin B, Lina G, Gocko X, Dieudonné M, Picone O, Bodaghi B, Gangneux JP, Degeilh B, Partouche H, Lenormand C, Sotto A, Raffetin A, Monsuez JJ, Michel C, Boulanger N, Cathebras P, Tattevin P; endorsed by scientific societies. Lyme borreliosis and other tick-borne diseases. Guidelines from the French Scientific Societies (I): prevention, epidemiology, diagnosis. Med Mal Infect. 2019 Aug;49(5):318-34. DOI: 10.1016/j.medmal.2019.04.381

Jaulhac B, Saunier A, Caumes E, Bouiller K, Gehanno JF, Rabaud C, Perrot S, Eldin C, de Broucker T, Roblot F, Toubiana J, Sellal F, Vuillemet F, Sordet C, Fantin B, Lina G, Sobas C, Gocko X, Figoni J, Chirouze C, Hansmann Y, Hentgen V, Cathebras P, Dieudonné M, Picone O, Bodaghi B, Gangneux JP, Degeilh B, Partouche H, Lenormand C, Sotto A, Raffetin A, Monsuez JJ, Michel C, Boulanger N, Lemogne C, Tattevin P; endorsed by scientific societies. Lyme borreliosis and other tick-borne diseases. Guidelines from the French scientific societies (II). Biological diagnosis, treatment, persistent symptoms after documented or suspected Lyme borreliosis. Med Mal Infect. 2019 Aug;49(5):335-46. DOI: 10.1016/j.medmal.2019.05.001

Gocko X, Lenormand C, Lemogne C, Bouiller K, Gehanno JF, Rabaud C, Perrot S, Eldin C, de Broucker T, Roblot F, Toubiana J, Sellal F, Vuillemet F, Sordet C, Fantin B, Lina G, Sobas C, Jaulhac B, Figoni J, Chirouze C, Hansmann Y, Hentgen V, Caumes E, Dieudonné M, Picone O, Bodaghi B, Gangneux JP, Degeilh B, Partouche H, Saunier A, Sotto A, Raffetin A, Monsuez JJ, Michel C, Boulanger N, Cathebras P, Tattevin P; endorsed by the following scientific societies. Lyme borreliosis and other tick-borne diseases. Guidelines from the French scientific societies. Med Mal Infect. 2019 Aug;49(5):296-317. DOI: 10.1016/j.medmal.2019.05.006

USA

Lantos PM, Rumbaugh J, Bockenstedt LK, Falck-Ytter YT, Aguero-Rosenfeld ME, Auwaerter PG, Baldwin K, Bannuru RR, Belani KK, Bowie WR, Branda JA, Clifford DB, DiMario FJ, Halperin JJ, Krause PJ, Lavergne V, Liang MH, Meissner HC, Nigrovic LE, Nocton JJJ, Osani MC, Pruitt AA, Rips J, Rosenfeld LE, Savoy ML, Sood SK, Steere AC, Strle F, Sundel R, Tsao J, Vaysbrot EE, Wormser GP, Zemel LS. Clinical Practice Guidelines by the Infectious Diseases Society of America (IDSA), American Academy of Neurology (AAN), and American College of Rheumatology (ACR): 2020 Guidelines for the Prevention, Diagnosis and Treatment of Lyme Disease. Clin Infect Dis. 2021 Jan 23;72(1):e1-e48. DOI: 10.1093/cid/ciaa1215

ILADS 2014

Cameron DJ, Johnson LB, Maloney EL. Evidence assessments and guideline recommendations in Lyme disease: the clinical management of known tick bites, erythema migrans rashes and persistent disease. Expert Rev Anti Infect Ther. 2014 Sep;12(9):1103-35. DOI: 10.1586/14787210.2014.940900

England

National Institute for Health and Care Excellence (NICE). Lyme disease. NICE guideline. NG95. NICE; 2018 Apr 11 [last updated 2018 Oct 17]. Available from: https://www.nice.org.uk/guidance/ng95

The Netherlands

Nederlandse Internisten Vereniging; Nederlandse Vereniging van Revalidatieartsen; Nederlandse Vereniging van Spoedeisende Hulp Artsen; Nederlandse Vereniging van Ziekenhuisapothekers; Nederlandse Vereniging voor Arbeids- en Bedrijfsgeneeskunde; Nederlandse Vereniging voor Cardiologie; Nederlandse Vereniging voor Dermatologie en Venereologie; Nederlandse Vereniging voor Kindergeneeskunde; Nederlandse Vereniging voor Klinische Chemie en Laboratoriumgeneeskunde; Nederlandse Vereniging voor Medische Microbiologie; Nederlandse Vereniging voor Neurologie; Nederlandse Vereniging voor Psychiatrie; Nederlandse Vereniging voor Reumatologie; Nederlandse Vereniging voor Verzekeringsgeneeskunde; Vereniging voor Infectieziekten, Sectie Infectieziektebestrijding. Richtlijn Lymeziekte. Utrecht: CBO; 2013. Available from: http://www.kiza.nl/sites/default/files/images/Richtlijn%20lymeziekte%20definitief%2018%20juli%202013.pdf

Annex 3: Overview of treatment recommendations for cutaneous Lyme borreliosis in international guidelines

**Guideline/treatment recommendation**	**Erythema migrans**	**Early disseminated form (without neurological manifestations)**	**Acrodermatitis chronica atrophicans (ACA)**
**USA**<br>Lantos et al. 2021 [215]	Doxycycline 100 mg 2x daily or 200 mg 1x daily for 10 days<br>Amoxicillin 500 mg 3x daily or<br>Cefuroxime axetil 500 mg 2x daily for 14 days or<br>azithromycin 500 mg daily for<br>5–10 days	Borrelia lymphocytoma<br>Doxycycline 100 mg 2x daily or 200 mg 1x daily for 21 days or amoxicillin 0.5–1 g 3x daily for 14 days or<br>cefuroxime axetil 500 mg 2x daily for 14–21 days<br>Lyme Karditis<br>Outpatients: Doxycycline 100 mg 2x daily or 200 mg daily<br>or<br>amoxicillin 500 mg 3x daily or cefuroxime axetil<br>500 mg 2x daily<br>Hospitalised patients: Initially ceftriaxone 2 g daily, then change to oral treatment for 14–21 days	Doxycycline 100 mg 2x daily or 200 mg 1x daily or<br>amoxicillin 0.5–1 g 3x daily or<br>cefuroxime axetil 500 mg 2x daily for 21–28 days
**Belgium**<br>Belkhir et al. 2016	Doxycycline 100 mg 2x daily for 10 days,<br>amoxicillin 500 mg 3x daily for 14 days,<br>Cefuroxime axetil 500 mg 2x daily for 14 days,<br>azithromycin 1 g on day 1, 500 mg on days 2–40<br>or 500 mg for 7 days, clarithromycin 500 mg 2X daily for 14 days	Lyme carditis:<br>Doxycycline 100 mg 2x daily for 21 days or ceftriaxone 2 g 1x daily for 14 days	Doxycycline 100 mg 2x daily for 21–28 days or ceftriaxone 2 g 1x daily for 14 days
**Germany**<br>**AWMF S1**<br>Hofmann et al. 2009 [217]	Doxycycline 100 mg 2x daily or amoxicillin 500–1,000 mg 3x daily or cefuroxime axetil 500 mg 2x daily for 14–21 days or<br>azithromycin: 250 mg 2x daily for 5–10 days		Doxycycline 100 mg 2x daily for 28 days or<br>ceftriaxone 2 g/daily or<br>cefotaxime 2 g 3x daily or<br>penicillin G $20x10^6$ U/daily for 14–21 days
**Denmark**	Penicillin V tablets 1.5 MIU x3 for 10 days (Children: 0.15 MIU/kg per day)<br>Alternatives if penicillin allergy:<br>Doxycycline tablet 200 mg on day 1, then 100 mg x1 for a total of 10 days (children over 12 years) same dose)<br>If penicillin allergy in pregnant patients and children under 12:<br>Cefuroxime axetil 500 mg x2 for 10 days (children: 30 mg/kg per day)	Penicillin V 1.5 MIU 3x daily for 21 days (children: 0.15 MIU/kg per day for 21 days)<br>Doxycycline 100 mg 2x daily for 21 days (same dose for children over 12)	Penicillin V 1.5 MIU 3x daily for 21 days (children: 0.15 MIU/kg per day for 21 days)<br>Doxycycline 100 mg 2x daily for 21 days (same dose for children over 12)

Attachment 1 to: *Hofmann H, Fingerle V, Rauer S, Hunfeld KP, Huppertz HI, Krause A, Consensus group. Cutaneous Lyme borreliosis: Guideline of the German Dermatology Society. GMS Ger Med Sci. 2025;23:Doc12.*
DOI: 10.3205/000348, URN: urn:nbn:de:0183-0003487

Guideline/treatment recommendation	Erythema migrans	Early disseminated form (without neurological manifestations)	Acrodermatitis chronica atrophicans (ACA)
**France**<br>Jaulhac et al. 2019 [213]	Doxycycline 100 mg 2x daily or amoxicillin 1 g 3x daily for 14 days	Lymphocytoma:<br>Doxycycline 200 mg/daily or amoxicillin 1 g 3x daily for 21 days	Doxycycline 200 mg/daily or ceftriaxone 2 g/daily for 28 days
**Netherlands**<br>Nederlandse Internisten Vereniging et al. 2004	Doxycycline 100 mg 2x daily for 10 days or amoxicillin 500 mg 3x daily for 14 days or azithromycin 500 mg/daily for 5 days	Doxycycline 100 mg 2x daily for 21 days or ceftriaxone 2 g/daily for 14 days	Doxycycline 100 mg 2x daily<br>(30 days)
**France**<br>Haute Autorité de santé (HAS). Borréliose de Lyme et Autres Maladies Vectorielles à Tiques (MVT). 2018. p. 1-26.	Doxycycline 100 mg 2x daily or 200 mg 1x daily or amoxicillin 1 g 3x daily for 14 days<br>Azithromycin 1 g on day 1; then 500 mg daily for 6 days	Doxycycline 100 mg 2x daily or<br>200 mg 1x daily or amoxicillin 1–2 g 3x daily for 21 days<br>Azithromycin 1 g on day 1; then 500 mg daily for 9 days	Doxycycline 200 mg or<br>ceftriaxone 2 g for 28 days
**Poland:**<br>Pancewicz, Garlicki, et al. 2015 [218]	Doxycycline 100 mg 2x daily or cefuroxime axetil 500 mg 2x daily or amoxicillin 500 mg 3x daily for 14–28 days	Borrelial lymphocytoma:<br>Doxycycline 100 mg 2x daily or cefuroxime axetil 500 mg 2x daily or amoxicillin 1.5–2 g daily for 14–28 days	Doxycycline 100 mg 2x daily or ceftriaxone 2/daily or<br>amoxicillin 1.5–2 g daily or cefuroxime axetil 500 mg 2x daily for 14–21 days
**England**<br>National Institute for Health and Care Excellence (NICE) 2018	Doxycycline 100 mg 2x daily or<br>200 mg 1x daily or<br>amoxicillin 1 g 3x daily for 21 days<br>or<br>azithromycin<br>500 mg daily for 17 days		Doxycycline 100 mg 2x daily or<br>200 mg 1x daily<br>or<br>amoxicillin 1 g 3x daily<br>or<br>Ceftriaxone 2 g daily for 28 days
**Finland**<br>Oksi et al. 2008	Amoxicillin 500–1,000 mg 3x daily or doxycycline 100 mg 2x daily or cefuroxime axetil 500 mg 2x daily for 14 days or azithromycin 500 mg/daily for 10 days	-	Ceftriaxone 2 g/daily for 14–21 days or amoxicillin 500 mg 3x daily or doxycycline 100 mg 2x daily for 1–2 months
**Norway**<br>Ljøstad and Mygland 2009	Doxycycline 100 mg 2x daily or<br>200 mg/daily or amoxicillin 500 mg 3x daily for 14 days	Doxycycline 100 mg 2x daily for 14 days	Doxycycline 200 mg/daily or 100 mg 2x daily for 20 days
**Switzerland**<br>Evison et al. 2006 [209]	Doxycycline 100 mg 2x daily for 10 days or amoxicillin 500 mg 3x daily or cefuroxime axetil 500 mg 2x daily or clarithromycin 500 mg 2x daily for 14–21 days or azithromycin 500 mg/daily for 7–10 days		-

Attachment 1 to: *Hofmann H, Fingerle V, Rauer S, Hunfeld KP, Huppertz HI, Krause A, Consensus group. Cutaneous Lyme borreliosis: Guideline of the German Dermatology Society. GMS Ger Med Sci. 2025;23:Doc12.*
DOI: 10.3205/000348, URN: urn:nbn:de:0183-0003487

Guideline/treatment recommendation	Erythema migrans	Early disseminated form (without neurological manifestations)	Acrodermatitis chronica atrophicans (ACA)
**Germany**<br>Deutsche Borreliose-Gesellschaft e.V. 2011	Doxycycline 400 mg/d<br>or<br>amoxycillin 3,000–6,000 mg/d<br>or<br>cefuroxime 2x500 mg/d<br>or<br>clarithromycin 500–1,000 mg/d<br>or<br>azithromycin 500 mg/3–4x/w<br>**for at least 4 weeks**	Ceftriaxone 2 g/d i.v.<br>or<br>Cefotaxime 2–3x4 g/d i.v.<br>or<br>minocycline 200 mg/d p.o.<br>**Duration according to clinical course. If ineffective, change antibiotic after 4 weeks**	
**USA**<br>Cameron et al. 2014 [216]	**Duration of treatment 10–21 days or duration of treatment 4–6 weeks (preferable)**<br><br>Amoxicillin 1,500–2,000 mg/d<br>Children: 50 mg/d spread over 3 doses of max 1,500 mg<br>Cefuroxime 2x500 mg/d<br>Children: 20–30 mg/d spread over 2 doses of max 1,000 mg<br>Doxycycline 2x100 mg<br>Children 8 years and older: 4 mg/kg/d spread over 2 doses of max. 200 mg<br>azithromycin 250–500 mg/d Children: 10 mg/kg on day 1, then 5–10 mg/kg/d max. 500 mg/d<br>**If persists:** Higher dosages as required, possibly also combined with intracellular antibiotics.<br>Repeat the 4–6 week treatment if symptoms persist, as with Lyme arthritis, ACA, late neuroborreliosis		If symptoms reappear or if symptoms progress after DD, repeat antibiotic treatment* with a new alternative antibiotic** or combination therapy<br>*Possibly also<br>–i.m. penicillin G 1.2–3.6 units/week or i.v. ceftriaxone 2 g/d<br>-Tetracycline: 1,000–1,500 mg spread over 3–4 doses<br>-(cefotaxime)<br>** Note: ILADS emphasises that no fixed treatment regimens can currently be established due to a lack of evidence. Duration and dosage depend, among other things, on disease severity and response to treatment.
**Germany**<br>Gaubitz et al. 2014 [219]	Doxycycline 100 mg 2x daily p.o.<br>or 200 mg 1x daily p.o.<br>or<br>amoxicillin 3 to 4x500–1,000 mg<br>or<br>cefuroxime 2x daily 500 mg p.o. for 10–21 days or<br>azithromycin 2x500 mg p.o. on day 1, then 1x500 mg p.o. for 5 days		Doxycycline 100 mg 2x daily p.o.<br>or 200 mg 1x daily p.o.<br>or<br>amoxicillin 3x500–1,000 mg<br>or<br>ceftriaxone 1x daily 2 g i.v.<br>for 21 (14–30) days

Attachment 1 to: *Hofmann H, Fingerle V, Rauer S, Hunfeld KP, Huppertz HI, Krause A, Consensus group. Cutaneous Lyme borreliosis: Guideline of the German Dermatology Society. GMS Ger Med Sci. 2025;23:Doc12.*
DOI: 10.3205/000348, URN: urn:nbn:de:0183-0003487

Annex 4: Legal regulations

Duty to report

In Germany, there is no general obligation to report diseases or pathogens in accordance with the Infection Protection Act (IfSG).

In the **federal states of Bavaria, Berlin, Brandenburg, Mecklenburg-Western Pomerania, Rhineland-Palatinate, Saarland, Saxony, Saxony-Anhalt and Thuringia,** however, there are additional ordinances that extend the reporting obligations under the Infection Protection Act. According to these state regulations, the respective health authorities must be informed of any illness or death from Lyme borreliosis in the form of erythema migrans, acute neuroborreliosis and acute Lyme arthritis.

http://www.rki.de/DE/Content/Infekt/EpidBull/Merkblaetter/Ratgeber_LymeBorreliose.html

Occupational disease

The attending physician must immediately notify the accident insurance institution, e.g. the employers' liability insurance association, in accordance with Section 202 SGB VII if there is reasonable suspicion that Lyme borreliosis could be an occupational disease as per BK 3102. The description of this occupational disease is: diseases transmissible from animals to humans. As part of the assessment procedure, the accident insurance institution will arrange for or carry out the necessary occupational and medical examinations. Occupational groups at particular risk include forestry and woodland workers, gardeners, farmers and hunters [Triebig G, Kentner M, Schiele R. Arbeitsmedizin. 2nd ed. Stuttgart: Gentner-Verlag; 2008.]

Attachment 1 to: *Hofmann H, Fingerle V, Rauer S, Hunfeld KP, Huppertz HI, Krause A, Consensus group. Cutaneous Lyme borreliosis: Guideline of the German Dermatology Society. GMS Ger Med Sci. 2025;23:Doc12.*
DOI: 10.3205/000348, URN: urn:nbn:de:0183-0003487
