# Supplementary material for: Cutaneous Lyme borreliosis: Guideline of the German Dermatology Society
Source: Ger Med Sci. 2025 Oct 9;23:Doc12. doi: 10.3205/000348 (PMC12584192; doi:10.3205/000348)
Supplement: Competing interests [file GMS-23-12-s-002.pdf]

Attachment 2: Table for declaration of interests and management of conflicts of interest

The following table summarizes the declarations of interest, along with the results of the conflict of interest assessment and the measures adopted by the LL group after discussion of the issues and implemented during the consensus conference.

Guideline Coordination: Hofmann, Heidelore; Fingerle, Volker;

Guideline: Cutaneous Lyme Disease; Registry Number: 013/044; Class: S2k

	Work as a consultant and/or expert	Participation in an advisory board	Paid lecture/ training activities	Paid authorship or co-authorship	Research projects/ conducting clinical studies	Owner interests (patent, copyright, share ownership)	Indirect interests	Guideline topics affected by COI, classification regarding relevance, consequence
Prof. Dr. med. Bechter, Karl	Wellcome Trust London	Journal of Affective Disorders Reports, Elsevier Verlag	various scientific congresses	various scientific journals	EU study MOOD-STRATIFICATION, donation for research project CSF Studies by Dr. Hans Huber, Stuttgart	no	Member: of various scientific societies for psychiatric and psychoimmunological research, e.g., DGPPN, DGBP, EPA, ECNP, WPA; chair of the DGBP CSF Research Section, co-chair of the WPA Immunology Psychiatry Section, member of the board of trustees of the neuropsychiatric conference Mind INPC Pula. Scientific activities: psychiatric, particularly psychoimmunological research, review of submitted articles for various scientific journals, conference organization. Clinical activities: outpatient psychiatric/psychotherapeutic therapies. Participation in further education/ training: Psychoimmunology Expert Meetings for 20 years (see www.psychoimmunology-experts.de) Mind INPC Pula Congresses	none
Dr. jur. Breinlinger, Astrid	no	no	no	no	no	no	Member: Borreliose und FSME Bund Deutschland e.V. / Chairwoman	none
Dahlem, Ursula	no	no	no	no	no	no	Member: OnLyme-Aktion.org Aktionsbündnis gegen zeckenübertragene Infektionen Deutschland e.V. (Patients Association), elected representative and chairwoman	none

Attachment 2 to: Hofmann H, Fingerle V, Rauer S, Hunfeld KP, Huppertz HI, Krause A, Consensus group. Cutaneous Lyme borreliosis: Guideline of the German Dermatology Society. GMS Ger Med Sci. 2025;23:Doc12. DOI: 10.3205/000348, URN: urn:nbn:de:0183-0003487

	Work as a consultant and/or expert	Participation in an advisory board	Paid lecture/ training activities	Paid authorship or co-authorship	Research projects/ conducting clinical studies	Owner interests (patent, copyright, share ownership)	Indirect interests	Guideline topics affected by COI, classification regarding relevance, consequence
Dr. med. Fingerle, Volker	QCMD (Quality control for molecular diagnostics)	Pfizer	Bavarian State Office for Health and Food Safety Academy for Health and Food Safety – AGL, med update GmbH Infektio Update 2020 Wiesbaden, Junge DGHM/ Organization team specialist refresher course, Labor Dr. Fenner + Kollegen MVZ, Deutsche Gesellschaft für Tropenmedizin, Reisemedizin und Globale Gesundheit e.V. Specialist refresher course in microbiology, virology and infectious disease epidemiology, Symposion 2022 in Überlingen; MVZ Laborärzte Singen, 23. Annual meeting of the AG Dermatologische Infektiologie u. Tropendermatologie e. V., Scientific Meeting VI. Labuda's Days, 32nd ECCMID, Lisbon, Akademie	no	National Reference Center for Borrelia, Bavarian Health and Food Safety Authority	no	Member: Instand, Expert: Round robin testing for Borrelia PCR and serology, ESGBOR (ESCMID study group for Lyme borreliosis) steering committee, DGHM<br>scientific activity: Head of the German National Reference Centre for Borreliae, many different publications on e.g. Borrelia, Lyme Borreliosis, SARS-CoV-2 etc. z.B.: Borrelia Ecology and Evolution: Ticks and Hosts and the Environment, Epidemiological Surveillance of Lyme Borreliosis in Bavaria, Incidence of notified Lyme borreliosis in Germany, Guidelines for diagnosis and treatment in neurology – Lyme neuroborreliosis, Controversies in bacterial taxonomy: The example of the genus Borrelia, Zeckenassoziierte Erkrankungen, Borreliose, Lyme-Borreliose, Characteristics of Borrelia burgdorferi sensu lato, Laboratory Diagnosis of Lyme Borreliosis, Bavarian SARS-CoV-2-Public Health Laboratory Team. Comparison of nine different commercially available molecular assays for detection of SARS-CoV-2 RNA, Detection of the new SARS-CoV-2 variants of concern, SARS-CoV-2 Sentinel Surveillance in Primary Schools, Kindergartens, and Nurseries; and more…<br>Participation in further education/training:	None<br><br>briefly member of a pharmaceutical industry advisory board on vaccine development. Since no vaccine is currently approved or available for use in humans, it is not a topic of the LL

	Work as a consultant and/or expert	Participation in an advisory board	Paid lecture/ training activities	Paid authorship or co-authorship	Research projects/ conducting clinical studies	Owner interests (patent, copyright, share ownership)	Indirect interests	Guideline topics affected by COI, classification regarding relevance, consequence
			f. Infektionsmedizin, QCMD: International Advisory Board Meeting 2023				Virtual ESGBOR – ICLB symposium | International Conference on Lyme Borreliosis and other Tick-borne diseases (iclb2022.org). Lead organiser., Tick Webinar 17 November 2021	
Prof. Dr. med. Freitag, Michael	no	DAK-Gesundheit Krankenversicherung	no	no	Innofonds Project HOMERN and Project KOPAL	no	member: DEGAM (General Medicine), guideline work SLK (Borreliose, asthma/COPD, Irritable bowel syndrome) wissenschaftliche Tätigkeit: Borreliose, Nursing home residents, emergency room, On-call duty, antibiotics, prostate cancer screening, gout. Clinical activity: General medicine	none
PD Dr.med. Gossrau, Gudrun	Social Court, Saxony Police	Novartis, Lilly, Teva, Lundbeck	Saxon State Medical Association, Novartis, Lilly, Teva, no	Teva	Novartis	no	Member: German Migraine and Headache Society, Executive Board Member Scientific Activity: German Pain Society, Member of the Children's Pain Working Group Clinical activity: International Headache Society, Member Participation in further education/training: DGN, Member	none
Prof. Dr. med. Gerd E. Gross	no	no	no	no	no	no	no	none
Prof. Dr. med. Hausteiner-Wiehle, Constanze	DGUV	no	CARUS Qualification Program for Clinical Research, Psychosomatic Clinic Windach	Deutsche Medizinische Wochenschrift, Deutsches Ärzteblatt, various specialist book publishers (Schattauer/Klett-Cotta, Elsevier), VG Wort	no	no	Member: German College for Psychosomatic Medicine DKPM and German Society for Psychosomatic Medicine and Medical Psychotherapy DGPM: Steering group AWMF guideline "Functional Body Complaints" (with patient guideline), author AWMF guideline "Cuta-	none

	Work as a consultant and/or expert	Participation in an advisory board	Paid lecture/ training activities	Paid authorship or co-authorship	Research projects/ conducting clinical studies	Owner interests (patent, copyright, share ownership)	Indirect interests	Guideline topics affected by COI, classification regarding relevance, consequence
							neous Lyme Borreliosis"; Working Group on Functional Neurological Disorders (member and board), Journal of Psychosomatic Research (Editorial Board) Scientific and clinical activities: Consultation and liaison psychosomatics, functional physical complaints	
Prof. Dr. med. Hofmann, Heidelore	Infectopharm, Report for the arbitration board of the medical associations	no	no	no	no	no	Member: German Dermatological Society, Working Group on Dermatological Infectious Diseases, Working Group on Pediatric Dermatology Clinical activity: Vaccination doctor for SARS-CoV-2 vaccination	Contract fees from the pharmaceutical industry on a topic of LL<br>low<br>none
Prof. Dr. med. Hunfeld, Klaus-Peter	Roche, Diasorin	Instand	no	Springer	no	no	Member: DGHM, DGKL, PEG, BÄMI, Instand<br>Scientific activities: vector-borne pathogens, public health, sepsis, microbiological diagnostics Clinical activities: laboratory medicine and microbiology, hospital hygiene at the hospital	None<br>Consulting/expert activities for the pharmaceutical industry: Specific/ commercial tests, including those for Lyme disease diagnostics, are neither discussed nor evaluated/ recommended in the LL; therefore, no restrictions apply.
Prof. Dr. med. Huppertz, Hans-Iko	no	Pfizer, GSK, Biontech	no	no	no	no	Member: German Academy of Pediatric and Adolescent Medicine, Alliance for Child and Adolescent Health, Commissioner for Children's Rights. Academic activities: Pediatric infectious diseases, rheumatology, immunology. Clinical activities: General pediatrics, rheumatology, immunology, infectious diseases.	none

	Work as a consultant and/or expert	Participation in an advisory board	Paid lecture/ training activities	Paid authorship or co-authorship	Research projects/ conducting clinical studies	Owner interests (patent, copyright, share ownership)	Indirect interests	Guideline topics affected by COI, classification regarding relevance, consequence
PD Dr. med. Kastenbauer, Stefan	no	no	no	no	no	no	scientific activity: Neuroinfectious diseases; clinical activity: Neurological practice	none
Prof. Dr. Kopp, Ina	German Accreditation Body (DAkkS) German Accreditation Body (DAkkS)	Institute for Quality Assurance and Transparency in Health Care (IQTIG), Medical Center for Quality in Medicine (ÄZQ)	EBM Frankfurt, AG of the Institute of General Medicine at the Faculty of Medicine of the Johann Goethe University Frankfurt, European Federation of Periodontology, European Society of Endodontology (ESE), German Society for Pediatric Infectious Diseases (DGPI)/ German Academy for Child Development and Health, European Business School (EBS) of the University of Economics and Law gGmbH, British Society for Periodontology (BSP), European Society for Contact Dermatitis (ESCD), Federal Association of Implantological Dentists in Europe (BDIZ)	VG-Wort	German Cancer Aid Foundation (DKH), Federal Ministry of Health (BMG), Federal Joint Committee (G-BA), Innovation Fund, Federal Ministry of Education and Research (BMBF)	no	Member: Steering Committee for the Oncology Guidelines Program of the German Cancer Society, German Cancer Aid and AWMF, Standing Commission on Guidelines of the AWMF (Deputy Chair), Primary Contact on behalf of the AWMF in the Guidelines International Network, German Network for Evidence-Based Medicine, German Society for Surgery, Advisory Board for the National Care Guidelines Program of the German Medical Association, National Association of Statutory Health Insurance Physicians and AWMF, Cohort 1: SCIANA-Health Leaders Network, funded by the Robert Bosch Foundation, Health Foundation, Careum Foundation, Board of Trustees of the Institute for Quality Assurance and Transparency in Health Care (IQTIG), German Society for Senology<br>Scientific activity: Guidelines, quality management, health services research Participation in further education/training: Seminars on guidelines of the AWMF for guideline developers and the curriculum guideline consultants, Methods workshops of the Oncology Guideline Program	none

	Work as a consultant and/or expert	Participation in an advisory board	Paid lecture/ training activities	Paid authorship or co-authorship	Research projects/ conducting clinical studies	Owner interests (patent, copyright, share ownership)	Indirect interests	Guideline topics affected by COI, classification regarding relevance, consequence
Prof. Dr. Krause, Andreas	BMS, Valneva/Pfizer	AbbVie, Amgen BMS, Boehringer Ingelheim, Gilead Janssen, Lilly, MSD, Mylan, Novartis, Pfizer, Sanofi	AbbVie, Amgen, Berlin Chemie, BMS, Boehringer Ingelheim, Gilead, Janssen, Lilly, Medac, MSD, Novartis, Pfizer, Roche, Sanofi, UCB	Boehringer Ingelheim	AbbVie, Lilly, Novartis,	no	Member: Board of the German Society for Rheumatology and Clinical Immunology, Board of the Association of Rheumatological Acute Clinics, Professional Association of German Rheumatologists, German Rheumatology League, German Society for Internal Medicine,<br>scientific activities: Lung involvement in rheumatic diseases, care,<br>clinical activities: Internal Medicine, Rheumatology	none
Prof. Dr. med. Müller, Rainer	no	no	no	no	no	no	Member: DGHNO, DGPP<br>Clinical activity: Consultation ENT, Phoniatrics and Paedaudiology consultation	none
Pauschinger, Matthias	no	no	no	no	no	no	no	none
Prof. Dr. med. Rauer, Sebastian	Roche Pharma AG, Novartis Pharma GmbH,	Roche Pharma AG, Novartis Pharma GmbH, Bristol-Myers	Roche Pharma AG, Novartis Pharma GmbH	Thieme Verlag Stuttgart	Novartis Pharma GmbH, Roche Pharma AG,	Co-owner (50%) of ravo Diagnostika GmbH	Member: DGN scientific activity: German Society for CSF Diagnostics and Clinical Neurochemistry (DGLN) c/o Kornelia Hauser, Department of Neurology, University Hospital Ulm, Neuroimmunology, Neuroinfectiology, General Neurology	Member Data Safety Board Member (DSMB) for Lyme vaccine development -> vaccination not part of the current guideline, co-owner of a company producing serological tests for the diagnosis of Lyme disease -> specific tests are not discussed in the guideline<br>Moderate<br>No voting rights<br>Note: Co-coordinator without thematically relevant conflicts of interest in the

	Work as a consultant and/or expert	Participation in an advisory board	Paid lecture/ training activities	Paid authorship or co-authorship	Research projects/ conducting clinical studies	Owner interests (patent, copyright, share ownership)	Indirect interests	Guideline topics affected by COI, classification regarding relevance, consequence
								steering group:<br>Mr. Kastenbauer
Prof. Rieger, Monika A.	no	no	no	no	no, funded by BMG, funded by the Ministry of Science, Research and the Arts Baden-Württemberg	no	Member: Guideline representative of the board of the DGAUM, mandate holder of the DGAUM in the guideline Cutaneous Lyme disease and in the guideline Neuroborreliosis, mandate holder of the DGAUM in the general assembly of the German Network for Health Services Research<br>Scientific activity: occupational health services research and use of occupational physiological methods to clarify work-related stress and design “good work”<br>Clinical activity: occupational medical care for various companies and institutions, in particular University of Tübingen<br>personal relationship: 1 brother is a lawyer at Allianz Private Health Insurance	none
Dr. med. Dr. med. dent. Rixecker, Herbert	no	no	no	no	no	no	Member: German Society for Oral and Maxillofacial Surgery, German Society for Implantology, German Society for Dentistry, Oral and Maxillofacial Surgery, Member and Chairman of the German Lyme Disease Society,<br>Clinical Practice: Oral and Maxillofacial Surgery	none

	Work as a consultant and/or expert	Participation in an advisory board	Paid lecture/ training activities	Paid authorship or co-authorship	Research projects/ conducting clinical studies	Owner interests (patent, copyright, share ownership)	Indirect interests	Guideline topics affected by COI, classification regarding relevance, consequence
Prof. Dr. med. Salzberger, Bernd	no	no	no	no	no	no	Member: German Society for Infectious Diseases, 1st Chair/ Board Scientific activities: Viral infections (HIV, CMV, influenza, SARS-CoV-2) Clinical activities: Treatment of patients with infections, including Lyme disease	none
Prof. Dr. rer. nat. Wallich, Reiner	no	no	no	no	no	no	no	none
Dr. med. Wilking, Hendrik	Federal Ministry of Health	no	no	no	no	no	Scientific activity: Infectious disease epidemiology, Public Health<br>Participation in further education/training: Lead teaching of infectious disease epidemiology at the Charité Berlin	none
